# Pro-Inflammatory Cytokine Priming and Purification Method Modulate the Impact of Exosomes Derived from Equine Bone Marrow Mesenchymal Stromal Cells on Equine Articular Chondrocytes

**DOI:** 10.3390/ijms241814169

**Published:** 2023-09-16

**Authors:** Manon Jammes, Frédéric Cassé, Emilie Velot, Arnaud Bianchi, Fabrice Audigié, Romain Contentin, Philippe Galéra

**Affiliations:** 1 BIOTARGEN, UNICAEN, Normandie University, 14000 Caen, France; manon.jammes@unicaen.fr (M.J.); frederic.casse@unicaen.fr (F.C.); romain.contentin@unicaen.fr (R.C.); 2 Molecular Engineering and Articular Physiopathology (IMoPA), French National Center for Scientific Research (CNRS), Université de Lorraine, 54000 Nancy, France; emilie.velot@univ-lorraine.fr (E.V.); arnaud.bianchi@univ-lorraine.fr (A.B.); 3 Center of Imaging and Research in Locomotor Affections on Equines, Veterinary School of Alfort, 14430 Goustranville, France; fabrice.audigie@vet-alfort.fr

**Keywords:** osteoarthritis, cartilage, chondrocytes, mesenchymal stromal cells, priming, cytokines, exosomes, regenerative therapy, horse

## Abstract

Osteoarthritis (OA) is a widespread osteoarticular pathology characterized by progressive hyaline cartilage degradation, exposing horses to impaired well-being, premature career termination, alongside substantial financial losses for horse owners. Among the new therapeutic strategies for OA, using mesenchymal stromal cell (MSC)-derived exosomes (MSC-exos) appears to be a promising option for conveying MSC therapeutic potential, yet avoiding the limitations inherent to cell therapy. Here, we first purified and characterized exosomes from MSCs by membrane affinity capture (MAC) and size-exclusion chromatography (SEC). We showed that intact MSC-exos are indeed internalized by equine articular chondrocytes (eACs), and then evaluated their functionality on cartilaginous organoids. Compared to SEC, mRNA and protein expression profiles revealed that MAC-exos induced a greater improvement of eAC-neosynthesized hyaline-like matrix by modulating collagen levels, increasing PCNA, and decreasing Htra1 synthesis. However, because the MAC elution buffer induced unexpected effects on eACs, an ultrafiltration step was included to the isolation protocol. Finally, exosomes from MSCs primed with equine pro-inflammatory cytokines (IL-1β, TNF-α, or IFN-γ) further improved the eAC hyaline-like phenotype, particularly IL-1β and TNF-α. Altogether, these findings indicate the importance of the exosome purification method and further demonstrate the potential of pro-inflammatory priming in the enhancement of the therapeutic value of MSC-exos for equine OA treatment.

## 1. Introduction

Osteoarthritis (OA) is a cartilage degenerative disease that affects both humans and animals. In horses, OA is a major cause of lameness and inevitably leads to decreased joint functions, animal welfare concerns, and increased risk of injury [1]. OA can occur due to aging, although this disease is also associated with athletic activity and can affect horses of any age, prematurely ending their sporting career and resulting in tremendous economic consequences. OA affects the whole articulation and is primarily characterized by a progressive breakdown of hyaline articular cartilage, inflammation, swelling, subchondral bone exposure, and pain. Furthermore, inflammation plays a significant role in OA physiopathology, manifested by the production of pro-inflammatory cytokines, which in turn contribute to cartilage degradation and joint pain. Hyaline articular cartilage is a connective tissue that is essential for proper joint functioning, protecting the bone ends from shocks and facilitating joint mobility. These properties directly arise from the composition of its cartilaginous extracellular matrix (ECM). Synthesized by chondrocytes, the cartilage ECM is made up of proteoglycans, which favor water retention, and collagen fibers, which promote elasticity and resistance to mechanical stress [2]. During OA, cartilage repair is limited by the poor self-regenerative potential of cartilage tissue, inducing a shift in the cartilage composition from a hyaline ECM rich in type IIB collagen to a fibrotic ECM with a higher amount of type I collagen. This neosynthesized fibrocartilage does not have the same properties as hyaline cartilage and is more susceptible to damage and degradation, which can even accelerate the progression of OA [3].

OA is driven by complex molecular and cellular interactions that are not completely understood, making management of the disease difficult and prompting ongoing research efforts. To date, the only OA symptom-relieving treatments available are nonsteroidal anti-inflammatory drugs (NSAIDs), analgesics, intra-articular injections of hyaluronic acid (HA), physical therapy, assistive devices, or surgery. These methods aim to relieve pain, reduce inflammation, improve joint mobility and muscle strength, and reduce stress on the joints [4]. However, none of these developments have ever reversed the course of OA and regenerated a fully functional hyaline cartilage.

Over the past few years, mesenchymal stromal cells (MSCs) have been extensively studied for their potential in OA management. Present in various tissues throughout the organism—e.g., bone marrow, adipose tissue, umbilical cord blood, dental pulp, etc.—MSCs are able to self-replicate and can differentiate into adipocytes, osteoblasts, and chondrocytes [5]. Their anti-inflammatory and immunomodulatory properties make them an appealing candidate to reduce OA-related inflammation and therefore prevent further damage to the joint. Moreover, MSCs have shown potential in promoting tissue regeneration, which is currently being investigated to improve the management of several affections, including equine OA [6]. Administered through intra-articular injections, equine MSCs (eMSCs) have demonstrated the ability to reduce pain and increase cartilage repair [7,8,9,10] while improving clinic symptoms and athletic performance [11,12,13]. Although MSC-based therapies hold significant promise, there are potential risks associated with their use, including immunogenicity, cell death at the site of injection, cell migration [14,15,16,17,18], and a possibility of tumoral transformation inherent to cell therapy, even though this latter risk is a subject of debate in the scientific community [19,20].

Recent research has highlighted that MSC therapeutic potential is partially driven by their secretome, which refers to all the soluble bioactive elements and particles released by the cells into the surrounding environment. The secretome includes extracellular vesicles (EVs), small membrane-bound structures categorized into apoptotic bodies, microvesicles, and exosomes according to their size and biogenesis pathway. For example, MSC-derived conditioned media (MSC-CM) have shown promising abilities to increase equine articular chondrocyte (eAC) migration and collagen accumulation in vitro [21]. These effects may be mediated by EVs, because intact, nano-sized, round-shaped vesicles have been observed in MSC-CM with transmission electron microscopy (TEM). However, the secretome of MSCs is not constant and can be influenced by various extrinsic parameters such as oxic conditions, the presence of inflammatory factors, or mechanical stimulation [22]. Among these methods, pro-inflammatory cytokines hold great promise in stimulating MSC therapeutic properties because MSCs are sensitive to immune stimulation, leading to the modulation of their secretome [23]. Recently, our research group highlighted the potential of interleukin 1β (IL-1β), tumor necrosis factor α (TNF-α), and interferon γ (IFN-γ) in promoting the ability of MSC-CM to significantly regenerate cartilage tissue [24]. Overall, cytokine priming triggers the stimulation of the secretion of immunomodulatory mediators in MSCs, enhances the chondroprotective effects of the MSC secretome and downregulates cartilage catabolism-associated markers such as matrix metalloproteases (MMPs) [22]. However, the use of purified exosomes has been found to offer greater safety and standardization advantages than the use of the entire MSC secretome [25]. Additionally, exosome membranes protect their cargo from environmental stress, while their surface proteins facilitate targeting of specific cells or tissues, which contribute to their potential as a more efficient and targeted therapeutic approach for specific applications [26,27]. MSC-derived exosomes (MSC-exos) show great potential as an OA-relieving treatment by upregulating cartilage anabolism-associated markers, like type II collagen and aggrecan, while decreasing catabolic, inflammatory, and apoptosis marker expression (for review [28]).

To harness the potential of exosomes, many exosome isolation methods are available and experimental protocols must be carefully established to ensure consistent results. Differential ultracentrifugation (dUC) stands out as the gold standard for exosome isolation; however, this method suffers from several limitations such as contaminant co-isolation, particle aggregation, risk of damaging exosomes, time-consuming protocols, or requiring expensive equipment [29]. Thus, more and more studies have considered switching to alternative purification methods. For example, polyethylene glycol (PEG) precipitation inevitably leads to the co-isolation of undesired particles and is therefore incompatible with in vivo applications. Additionally, the contamination of exosome samples with PEG can restrict the use of additional analyses such as mass spectrometry [30]. However, the large quantity of collected EVs makes PEG precipitation appropriate for efficient marker detection. During the last few years, a considerable amount of effort has been made to develop isolation protocols combining satisfactory yield and purity levels. Among them, size-exclusion chromatography (SEC) has emerged as a promising method for exosome isolation, providing high purity samples suitable for in vivo approaches [31]. Approved for a large panel of biological fluids, SEC generally prevents exosome deformation and break-up, which occur more often with other isolation methods such as ultrafiltration (UF) or dUC [32,33]. Nonetheless, despite its multiple advantages, SEC shows one of the lowest yields among the exosome purification methods, which complexifies its application to large-scale utilization. Concomitantly, membrane affinity capture (MAC) has also proven its effectiveness for exosome purification from various biological fluids [29]. Based on a membrane used to bind and selectively isolate exosomes, MAC can recover a significant proportion of the exosomes present in a biological sample. This method is advantageous due to its relative speed and ease of use, and provides samples of intermediate purity. Overall, when choosing an isolation technique, it is important to strike a balance between yield and purity that aligns with the nature of the samples and the desired purpose of the exosome collection.

Following up on our previous work [21,22,24], in this study we aimed to investigate the presence of exosomes in MSC-CM and their implication in the effects attributed to the MSC secretome on equine articular chondrocyte (eAC) phenotype. For this purpose, we isolated exosomes using different isolation methods—i.e., PEG precipitation, MAC and SEC—whose capacities to generate consistent EV samples were compared using TEM, nanotracking analysis (NTA), and Western blotting. Then, to determine whether exosomes are beneficial for cartilage regeneration, collagen sponges seeded with eACs to generate a cartilaginous organoid were treated with exosomes isolated by either MAC (MAC-exos) or SEC (SEC-exos). Then, we analyzed the gene and protein expression patterns of typical and non-typical cartilage-associated markers to determine which approach was better adapted to preserve the therapeutic potential of MSC-exos. In addition, the exosome internalization by eACs was studied, in both bi-(2D) and tridimensional (3D) cultures, using fluorescent labeling of exosomes and confocal microscopy. Finally, exosomes derived from MSCs primed with either IL-1β, TNF-α, or IFN-γ were purified based on the previous results and the efficacy of each priming method in enhancing the eAC phenotype was assessed by culturing cartilage-like organoids with MSC-exos.

## 2. Results

### 2.1. The Precipitation, MAC, and SEC Isolation Methods Effectively Purify Exosomes

**Figure 1:**
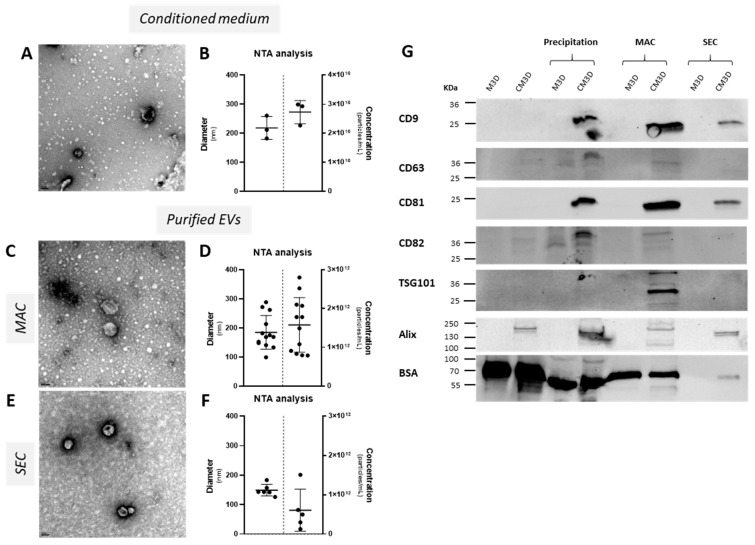
Purification methods can recover intact exosomes from CM. CM of equine BM-MSCs (P3) was collected after 24 h of culture and stored at −80 °C. EV morphology and integrity were checked by TEM ((**A**); scale bar: 100 nm). Nanoparticle concentration and size distribution were assessed using NTA on CM derived from three strains of BM-MSCs (**B**). Then, EVs were purified using PEG precipitation, the ExoEasy Maxi Kit (Qiagen, MAC method), or AFC (Izon Science, Christchurch, New Zealand, SEC method). EV morphology and integrity were checked with TEM (**C**,**E**); scale bar: 50 nm) (MAC: *n* = 3; SEC: *n* = 3). Nanoparticle concentration and size distribution were assessed using NTA on EVs derived from BM-MSC (**D**,**F**) (MAC: *n* = 13; SEC: *n* = 6). Mean values of NTA results are represented as scatter dot plots ± standard deviation. Finally, Western blots were used to assess the expression of several EV-associated proteins (**G**). BSA was used as a purity marker and M3D corresponds to unconditioned medium. BM-MSCs, bone marrow-mesenchymal stem cells; BSA, bovine serum albumin; CM, conditioned media; EVs, extracellular vesicles; NTA, nanoparticle tracking analysis; P3, passage 3; TEM, transmission electron microscopy.

First, TEM and NTA analyses were carried out to confirm the presence of exosomes in CM and purified samples. In filtered CM, round-shaped and undamaged, intact particles (Figure 1A) were detected with a mean diameter of 218 nm and a concentration of 2.73 × 10^10^ ± 3.99 × 10^9^ particles/mL (Figure 1B). After MAC purification, the same type of particle was detected (Figure 1C). However, we also noticed the presence of a high quantity of impurities (Figure S1). In addition, the particles collected by MAC exhibited a mean diameter of 184.97 nm and a mean concentration of 1.58 × 10^12^ particles/mL (Figure 1D).

The SEC purification method also allowed the collection of spherical particles (Figure 1E) with a lower mean diameter (149.22 nm) and concentration (6.10 × 10^11^ particles/mL) than with the MAC method (Figure 1D,F). Additionally, less debris was found in SEC-exos samples, simplifying the TEM observation and detection of putative round-shaped nanoparticles. However, other structures were noticeable, indicating that the preparation was not perfectly pure. The presence of a panel of several exosome markers was then assessed by Western blot in unconditioned medium (M3D), filtered CM (CM3D), or after PEG precipitation, MAC or SEC (Figure 1G). The surface markers cluster of differentiation (CD)9 and CD81 were detected only consecutively to CM3D purification, regardless of the purification method. CD63 and CD82 were also detected in exosome samples extracted from CM3D by precipitation and MAC, but not significantly in the SEC-purified samples. Unlike CD9 and CD81, M3D precipitated with PEG showed a faint signal for CD63 and CD82. Regarding intraluminal proteins, TSG101 was only detectable in MAC-purified CM3D. All CM3D conditions were positive for Alix, even the raw CM, with the highest intensity after PEG precipitation. Interestingly, this protein appeared as a doublet which showed slightly different molecular weights between raw CM3D/MAC and PEG precipitation/SEC. BSA, a main component of fetal calf serum (FBS) used to supplement culture medium, was used as an indicator of sample purity as recommended by the International Society for Extracellular Vesicles (ISEV) [34]. BSA amounts were the highest in raw M3D and CM3D, but still detected after PEG precipitation and MAC samples. BSA was not detected in SEC-purified M3D and only a slight signal was observed in SEC-purified CM3D. Therefore, and compared with precipitation and MAC, SEC led to the recovery of exosomes with the best purity. Overall, exosome markers were mostly and exclusively detected in CM3D after all three purification methods. Moreover, the exosome marker amounts were the highest for MAC purification and the lowest for SEC.

Altogether, these results confirmed the presence of exosomes in CM derived from equine BM-MSC cultures. The purification/concentration methods we used exhibited different levels of yield and purity. Even though particles with a diameter greater than that expected for exosomes were detected by NTA, the mode diameter corresponded to typical sizes associated with this type of particle (Figure 1B,D,F). During these experiments, CM were filtered through a 0.22 µm porous membrane intended to remove particles with a larger diameter. However, the lipid composition of EV membranes allows for a certain level of membrane deformability. Furthermore, there is an overlap in the diameter range between large exosomes and small microvesicles, suggesting the presence of EVs and particles other than exosomes. Nevertheless, considering the significant proportion of exosome-sized particles in our samples, we refer to the purified particles as “exosomes” rather than “EVs” hereafter.

### 2.2. Purified Exosomes Can Be Internalized in eACs

**Figure 2:**
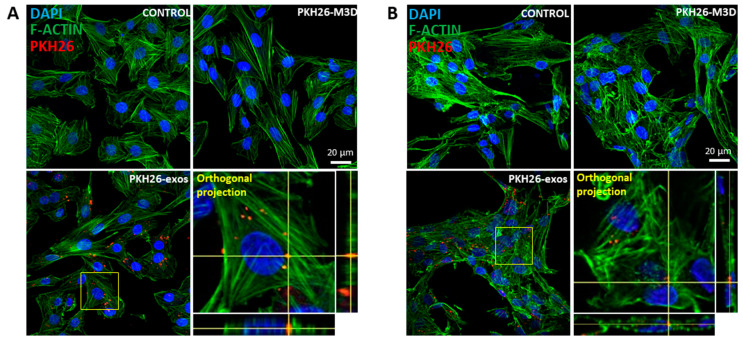
Exosomes derived from BM-MSCs are internalized by eACs cultured in monolayer or in collagen sponges. CM of equine BM-MSCs (P3) was collected after 24 h of culture. After centrifugation, filtration and concentration, samples were purified on a qEV35 column using the AFC (Izon Science) and reconcentrated. Exosomes were stained with the PKH26 Red Fluorescent Cell Linker Kit (Sigma-Aldrich, Saint Louis, MO, USA) and stored in PBS at −80 °C until use. eACs (P2) were seeded in monolayer (**A**) at 2 × 10^4^ cells/cm^2^ or in collagen sponges (**B**) at 800,000 cells/sponge, grown for 7 days under a hypoxic atmosphere and treated with 6 µg/mL of PKH26-labeled exosomes. After 24 h of culture, cells were fixed, labeled with DAPI and F-ACTIN, and imaged using a confocal microscope (yellow box: magnification; orthogonal projection depth: 30 µm). Control condition refers to untreated cells and M3D corresponds to eAC treated with exosomes purified from unconditioned medium. BM-MSCs, bone marrow-mesenchymal stem cells; CM, conditioned media; P2, passage 2; P3, passage 3.

To examine whether purified exosomes can be taken up by eACs, exosomes isolated from CM3D were labeled with PKH26, a lipophilic dye that can be integrated into the lipid bilayer, such as in the exosome membrane. The M3D underwent the same PKH26 labeling process as the CM3D and was used as the control. After 24 h or 48 h of exposition with EVs derived from CM3D (PKH26-exos), both 2D- and 3D-cultured eACs showed the PKH26 signal inside the cells, confirmed on the orthogonal projections, and suggesting the internalization of exosomes by eACs (Figure 2 and Figure S2, Video S1). Surprisingly, some PKH26 spots were noticeable in eACs cultured with M3D-derived exosomes (PKH26-M3D), which could correspond to remaining exosomes in the depleted FBS used for the cell culture or PKH26 micelles produced during the labeling process.

### 2.3. eACs Cultured with MAC-Exos Exhibit a Gene Expression Profile Closer to Hyaline Cartilage than eACs Cultured with SEC-Exos

**Figure 3:**
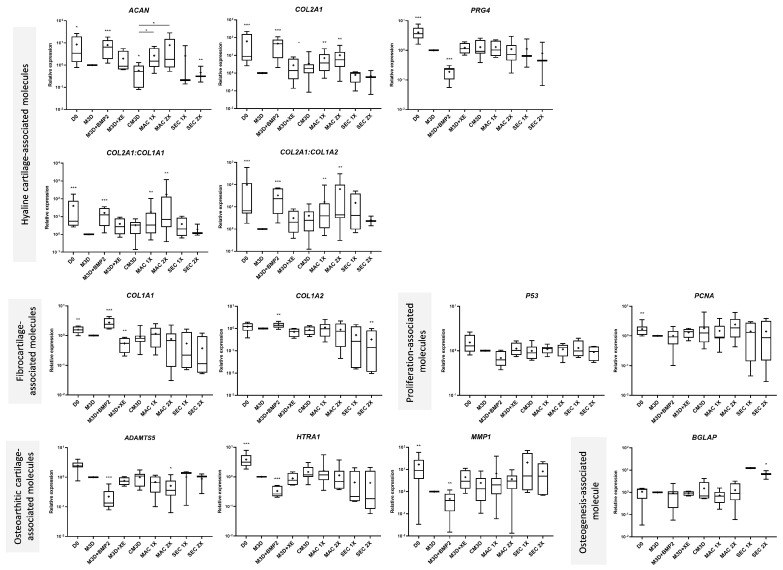
Purification method changes the effect of exosomes from BM-MSCs on the eAC gene expression of cartilage- and OA-associated markers. CMs from BM-MSCs (P3) were harvested and exosomes were isolated using either the MAC or the SEC method. Unconditioned medium (M3D) was aliquoted, supplemented with fresh exosomes, and stored at −80 °C. Controls were included by supplementing M3D with BMP2 (50 ng/mL) or Qiagen elution buffer (XE) and CM3D corresponds to BM-MSC culture medium conditioned for 24 h. The 1X and 2X concentrations refer to protein concentrations of exosome solution related to 1 mL of CM3D. eACs (P2) were seeded in collagen sponges at 800,000 cells/sponge and then cultured under hypoxic atmosphere with the different media for 14 days. Then, sponges were harvested, washed twice with PBS, and stored at −80 °C. Total RNA was collected from these cultures and RT-qPCRs were run to assess gene expression. The expression of target genes was normalized using either the reference genes *β-ACTIN* and *PPIA*. The D0 condition corresponds to eACs cultured in monolayer until P2. Experiments were reproduced with different strains of eACs and BM-MSCs (*n* = 8; *n* = 4 for Izon conditions). Values are represented as box plots (median, quartiles, extreme values, “+” corresponds to the mean) and tested using the Mann–Whitney test, * *p* < 0.05, ** *p* < 0.01, *** *p* < 0.005 significantly different from the M3D condition. BM-MSCs, bone marrow-mesenchymal stromal cells; BMP2, bone morphogenetic protein 2; CM, conditioned medium; eACs, equine articular chondrocytes; M3D, control medium with 2% FBS; P2, passage 2; P3, passage 3; RT-qPCR, reverse transcription-quantitative polymerase chain reaction; XE, Qiagen elution buffer.

The beneficial effect of MSC-CM on the eAC phenotype has been studied previously [21]. Herein, we sought to assess the effect of MSC-exos on eAC-derived organoids. Furthermore, considering the difference in sample yield and purity between MAC and SEC purifications, we also sought to analyze whether the purification method can lead to divergent effects on the eAC phenotype. We thus tested two different concentrations of exosomes using the MAC and SEC approaches on eACs cultured as organoids for 14 days. The 1X concentration corresponds to either 6 µg (MAC) or 0.5 µg (SEC) of total proteins per sponge. Controls were added to the conditions by adding BMP-2 (chondrogenic control) or Qiagen elution buffer (M3D + XE) to filtered M3D. For this latter condition, the volume of XE buffer added to M3D corresponds to the volume of the exosome solution added in the MAC 2X condition (specific to each strain and variable according to each EV purification method). In comparison with the M3D condition, *ACAN* levels of eACs cultured with CM3D were significantly lower. On the contrary, *COL2A1* mRNA levels seemed to be slightly higher (Figure 3). In accordance with a trend (apparent direction or movement in the data between two or more conditions but without reaching the significance threshold selected) to the decrease in both *COL1A1* (*p*-value = 0.0831) and *COL1A2* (*p*-value = 0.4042) fibrocartilage-associated markers, CM3D tended to increase the *COL2A1:COL1A1* and *COL2A1:COL1A2* functional ratios. Interestingly, the XE buffer displayed similar results for *COL2A1* and *COL1A1* mRNA levels, suggesting a potential interaction between this solution and eAC metabolism. We did not observe any changes in *P53* and *PCNA* transcript levels with CM3D, nor in the mRNA levels of *ADAMTS5*. Moreover, CM3D did not seem to modulate *HTRA1*, *MMP1*, *MMP13*, or *COL10A1* levels compared with eACs cultured with M3D. CM3D did not drastically modulate *BGLAP* and the inflammation-associated molecule *P65*. It is noteworthy that the elution buffer (XE) exhibited a tendency to increase *MMP1* and *MMP13* (*p*-value = 0.1879) mRNA levels.

Compared with M3D, MAC-exos initiated an increase in *ACAN* expression, which was significant when compared with CM3D. *COL2A1* mRNA levels increased in eACs cultured with MAC-exos, whereas *COL1A1* and *COL1A2* remained unchanged. Thus, both functional ratios increased significantly. Proliferation-associated *PCNA* and *P65* levels remained similar whether the eACs were cultured with M3D or with MAC-exos. Conversely, the highest concentration of MAC-exos triggered a significant decrease in *ADAMTS5* expression as well as in *P65*, compared with the M3D condition. The *HTRA1*, *MMP1*, and *BGLAP* mRNA levels did not decrease. MAC-exos tended to increase *COL10A1* levels, and *MMP13* mRNA levels remained unchanged compared to the M3D condition.

Regarding eACs cultured with the exosomes purified with the SEC method, they showed a decrease in *ACAN* mRNA levels, whereas the *COL2A1* and *PRG4* mRNA levels were slightly lower than those observed with eACs cultured with M3D. In addition, *COL1A1* and *COL1A2* decreased. Thus, the two functional ratios also tended to increase using SEC-exos. *PCNA* and *P53* mRNA levels of eACs cultured with SEC-exos remained unchanged. *ADAMTS5*, *HTRA1*, and *COL10A1* levels also remained similar whether the eACs were cultured with SEC-exos or M3D. On the contrary, eACs cultured with SEC-exos had higher *MMP1*, *MMP13*, and *BGLAP* mRNA levels. The levels of the inflammation-associated molecule, *P65*, also increased when eACs were cultured in the presence of SEC-exos, compared with eACs cultured with M3D or CM3D.

Altogether, our results indicate that MAC-exos favored the mRNA levels of hyaline cartilage-associated markers while decreasing the levels of *ADAMTS5* and *P65* (MAC-exos 2X). On the contrary, SEC-exos did not drastically modulate the levels of hyaline cartilage associated-markers, but decreased the type I collagen mRNA and increased the levels of *MMP1*, *MMP13*, *BGLAP*, and *P65*.

### 2.4. MAC-Exos Induce a Greater Cartilage-Associated Protein Expression Profile in eACs than SEC-Exos

**Figure 4:**
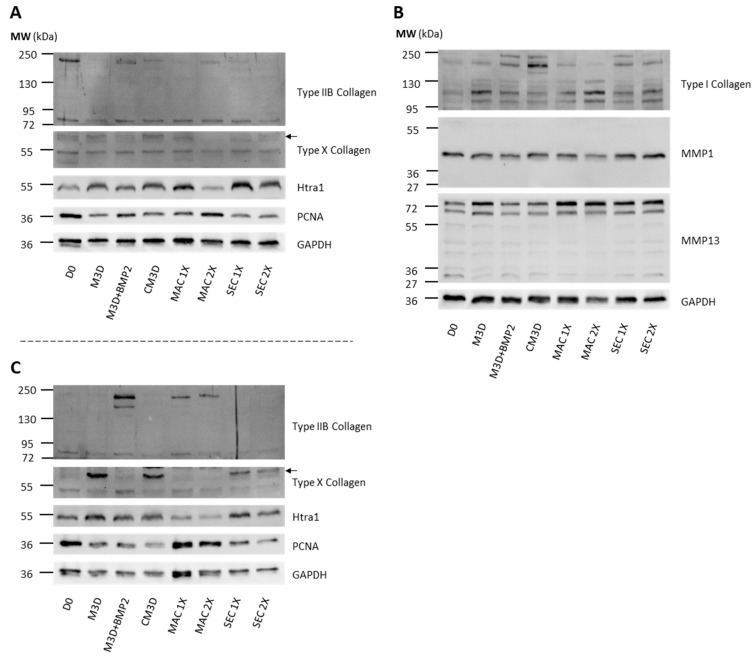
Purification method changes the effect of exosomes from BM-MSCs on the eAC expression of cartilage- and OA-associated proteins. CMs from BM-MSCs (P3) were harvested and exosomes were isolated using either the MAC or the SEC method. Unconditioned medium (M3D) was aliquoted, supplemented with fresh exosomes and stored at −80 °C. A chondrogenic control was included by supplementing M3D with BMP2 (50 ng/mL). CM3D corresponds to BM-MSC culture medium conditioned for 24 h. The 1X and 2X concentrations refer to protein concentrations of exosome solution related to 1 mL of CM3D. eACs (P2) were seeded in collagen sponges at 800,000 cells/sponge and then cultured under hypoxic atmosphere with the different media for 14 days. Then, sponges were harvested, washed twice with PBS, and stored at −80 °C. The total proteins were extracted using a dedicated buffer supplemented with protease inhibitors and Western blots were carried out to evaluate protein levels, as shown in the representative blots. The same protein targets are presented in subfigures (**A**,**C**), differing from blots presented in subfigure (**B**). The D0 condition corresponds to eACs cultured in monolayer until P3. Arrows indicate type X collagen bands. Experiments were reproduced with different strains of eACs and BM-MSCs (*n* = 4). BM-MSCs, bone marrow-mesenchymal stromal cells; CM, conditioned medium; eACs, equine articular chondrocytes; M3D, control medium with 2% FBS; P2, passage 2; P3, passage 3.

Then, we sought to determine if protein levels matched mRNA expressions. Western blots were performed on protein extracts from the same conditions as in RT-qPCR. Due to difficulties in presenting a single Western blot assay that represents all the experiments (*n* = 4), we opted to include two different assays for some of the protein blots to enhance their representativeness. Compared with eACs harvested before treatment (D0), type IIB collagen expression dropped in eACs cultured with CM3D (Figure 4A). The main effect of CM3D (compared with M3D) was the induction of collagen protein accumulation, with discrepancies between eAC strains regarding type IIB collagen (Figure 4A,C).

MAC-exos triggered an increase in type IIB collagen and PCNA protein accumulation, especially when 2X-concentrated MAC-exos were used, compared with M3D and CM3D conditions. On the contrary, the type I and type X collagens and Htra1 protein amounts were lower than in the M3D or CM3D conditions. Conversely, SEC-exos only slightly increased type IIB collagen protein accumulation compared with M3D (Figure 4A). Furthermore, type I collagen protein amounts remained lower than that obtained with CM3D, but increased in comparison with M3D, especially when eACs were cultured with the lowest SEC-exos concentration. Immunostaining of collagens provided further support, demonstrating an increase in type I collagen accumulation after a 24 h or 48 h incubation of eACs with MAC-exos, but not with SEC-exos (Figure S3). Nonetheless, we did not observe any variation in type II collagen expression between the control condition and after eAC treatment with MAC- or SEC-exos. Compared with the M3D and CM3D conditions, SEC-exos decreased type X collagen protein accumulation. Furthermore, PCNA protein amounts remained similar whether the eACs were cultured with SEC-exos or with M3D. The MMP1 protein amounts remained similar regardless of the conditions.

Altogether, our results demonstrate that MAC-exos show greater potential than SEC-exos for enhancing eAC ECM composition by improving type IIB and I collagen expression, and also by modulating type X collagen, Htra1, and PCNA levels. Regarding these considerations, we selected the MAC isolation method at the 2X concentration to further investigate the potential of exosomes to improve the eAC phenotype/cartilaginous organoid quality.

### 2.5. UF Does Not Improve MAC-Exos Potential for Enhancing eAC Phenotype

**Figure 5:**
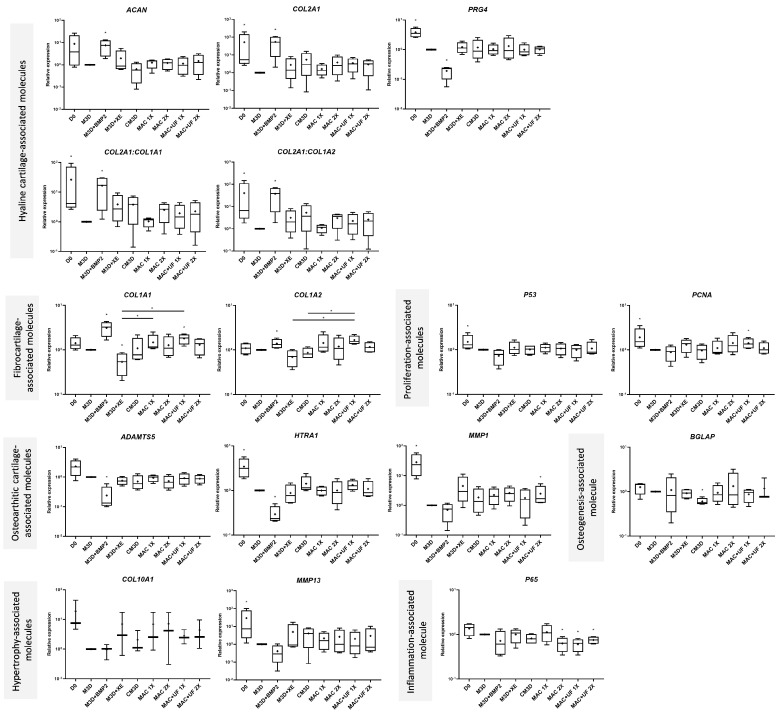
Ultrafiltration does not seem to improve the effect of exosomes isolated using the MAC method on eAC gene expression of cartilage- and OA-associated markers. CMs from BM-MSCs (P3) were harvested and exosomes were isolated using the MAC method followed or not by PBS washes through Amicon filtration (MAC + UF conditions). Unconditioned medium (M3D) was aliquoted, supplemented with fresh exosomes and stored at −80 °C. Controls were included by supplementing M3D with BMP2 (50 ng/mL) or Qiagen elution buffer (XE) and CM3D corresponds to BM-MSC culture medium conditioned for 24 h. The 1X and 2X concentrations refer to protein concentrations of exosome solution related to 1 mL of CM3D. eACs (P2) were seeded in collagen sponges at 800,000 cells/sponge and then cultured under hypoxic atmosphere with the different media for 14 days. Then, sponges were harvested, washed twice with PBS and stored at −80 °C. Total RNA was collected from these cultures and RT-qPCRs were run to assess gene expression. The expression of target genes was normalized using the reference genes *β-ACTIN* or *PPIA*. The D0 condition corresponds to eACs cultured in monolayer until P2. Experiments were repeated with different strains of eACs and BM-MSCs (*n* = 4). Values are represented as box plots (median, quartiles, extreme values, “+” corresponds to the mean) and were tested using the Mann–Whitney test, * *p* < 0.05 significantly different from the M3D condition. BM-MSCs, bone marrow-mesenchymal stromal cells; BMP2, bone morphogenetic protein 2; CM, conditioned medium; eACs, equine articular chondrocytes; M3D, control medium with 2% FBS; P2, passage 2; P3, passage 3; RT-qPCR, reverse transcription-quantitative polymerase chain reaction; XE, Qiagen elution buffer.

As suggested by the results of the previous RT-qPCR assays (Figure 3), the MAC elution buffer seemed to interfere with eAC metabolism. To prevent this inconvenience that could lead to a misevaluation of the therapeutic effect of MAC-exos, successive UF steps were performed to remove as much XE buffer as possible. The impact of MAC- and MAC + UF-exos at 1X (6 µg/sponge) and 2X (12 µg/sponge) concentrations was then investigated on eACs cultured as organoids for 14 days. As in previous experiments, the XE buffer added to M3D (M3D + XE) corresponds to the volume of MAC-exos added in the 2X condition for each strain. As observed in Figure 3, eACs cultured with elution buffer had similar *ACAN*, *COL2A1*, and *PRG4* mRNA levels, but decreased *COL1A1* and *COL1A2* (*p*-value = 0.3143) mRNA levels, compared with the M3D condition (Figure 5), resulting in higher *COL2A1:COL1A1* and *COL2A1:COL1A2* functional ratios. Additionally, eACs cultured with the XE buffer tended to have higher mRNA levels of *MMP1*, *MMP13*, and *COL10A1* compared with eACs cultured in M3D. 

**Figure 6:**
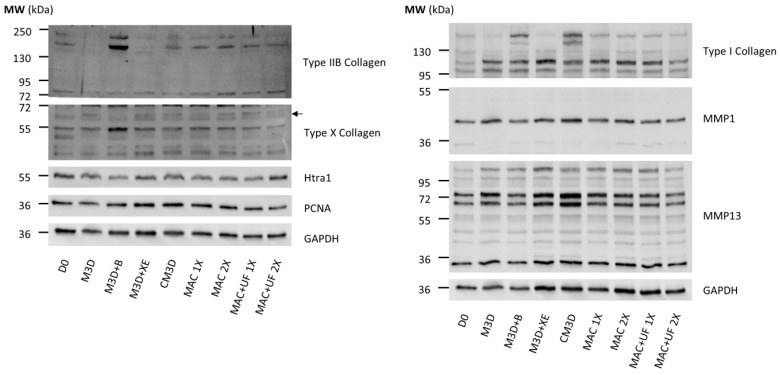
Ultrafiltration does not seem to improve the effect of exosomes isolated with MAC method on eAC expression of cartilage- and OA-associated proteins. CMs from BM-MSCs (P3) were harvested and exosomes were isolated using either the MAC or the SEC method. Unconditioned medium (M3D) was aliquoted, supplemented with fresh exosomes and stored at −80 °C. A chondrogenic control was included by supplementing M3D with BMP2 (50 ng/mL). CM3D corresponds to BM-MSC culture medium conditioned for 24 h. The 1X and 2X concentrations refer to protein concentrations of exosome solution related to 1 mL of CM3D. eACs (P2) were seeded in collagen sponges at 800,000 cells/sponge and then cultured under hypoxic atmosphere with the different media for 14 days. Then, sponges were harvested, washed twice with PBS, and stored at −80 °C. The total proteins were extracted using a dedicated buffer supplemented with protease inhibitors and Western blots were carried out to evaluate protein levels, as shown in the representative pictures. The D0 condition corresponds to eACs cultured in monolayer until P3. The arrow indicates type X collagen bands. Experiments were repeated with different strains of eACs and BM-MSCs (*n* = 4). BM-MSCs, bone marrow-mesenchymal stromal cells; CM, conditioned medium; eACs, equine articular chondrocytes; M3D, control medium with 2% FBS; P2, passage 2; P3, passage 3.

Overall, the UF steps did modify the effect of MAC-exos regarding the mRNA levels of the markers associated with hyaline cartilage and hypertrophy. Nevertheless, eACs cultured with 1X MAC-exos + UF had significantly increased levels of *COL1A1*, *PCNA*, and decreased levels of *P65*, compared with the M3D condition (Figure 5). The *COL1A1* and *COL1A2* mRNA levels were also higher than those in the M3D + XE condition. The eACs cultured with 2X MAC-exos + UF had significantly increased levels of *MMP1*, compared with M3D condition, even though the *MMP1* levels were similar among the four MAC-exos conditions. These differences in mRNA levels were not observed when eACs were cultured with 1X MAC-exos without the UF steps. Regarding type IIB collagen, type I collagen and type X collagen protein amounts, the effect of the MAC-exos on eACs was similar with or without UF (Figure 6). MMP1 and MMP13, Htra1, and PCNA protein amounts were also unaffected.

Overall, we did not observe any improvement in the effects induced by 2X MAC-exos on eAC protein and mRNA expression profiles after UF. Based on these results, we employed the original MAC purification protocol for the next part of the study, without the additional UF steps.

### 2.6. The Effect of Primed MSC-Derived Exosomes on eAC Gene Expression Is Masked by the Presence of the Elution Buffer

The preceding experiments allowed us to select an exosome isolation method—i.e., the MAC method represented in this study by the ExoEasy Maxi kit from the Qiagen company—that can provide rapidly purified MSC-exos with high yield and purity levels. Moreover, this purification strategy led to the recovery of exosomes demonstrating a promising potential for enhancing the eAC phenotype. However, the therapeutic potential of exosomes derived from naive BM-MSCs can be enhanced through various stimulation techniques, including the use of pro-inflammatory cytokines. In a previous study, we demonstrated that the priming of BM-MSCs with equine IL-1β, TNF-α, or IFN-γ can modulate the effects mediated by their secretome on cartilaginous organoids [24]. Here, we aimed to investigate whether exosomes isolated from primed BM-MSCs can also mediate similar effects. To do so, we primed naive BM-MSCs with cytokines for 24 h, and we collected their secretome over the same period. Then, we isolated the exosomes employing the MAC purification method. Afterward, cartilaginous organoids were cultured in M3D supplemented with either naive (naive-exos) or primed exosomes (IL1β-, TNFα-, or IFNγ-exos).

**Figure 7:**
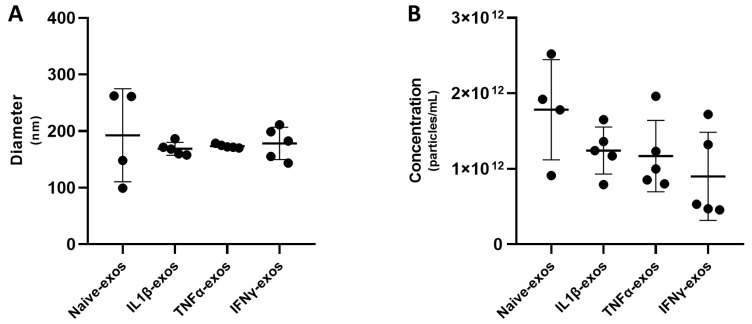
Pro-inflammatory priming homogenizes the diameter of MSC-derived particles while decreasing their concentration. After 24 h priming of BM-MSCs (P3) with either IL-1β, TNF-α, or IFN-γ, unconditioned medium (M3D) was conditioned for 24 h and exosomes were isolated from CMs using the MAC method. Size distribution (**A**) and nanoparticle concentration (**B**) were assessed using NTA. Mean values are represented as scatter dot plots ± standard deviation. BM-MSCs, bone marrow-mesenchymal stem cells; CM, conditioned media; EVs, extracellular vesicles; NTA, nanoparticle tracking analysis; P3, passage 3.

**Figure 8:**
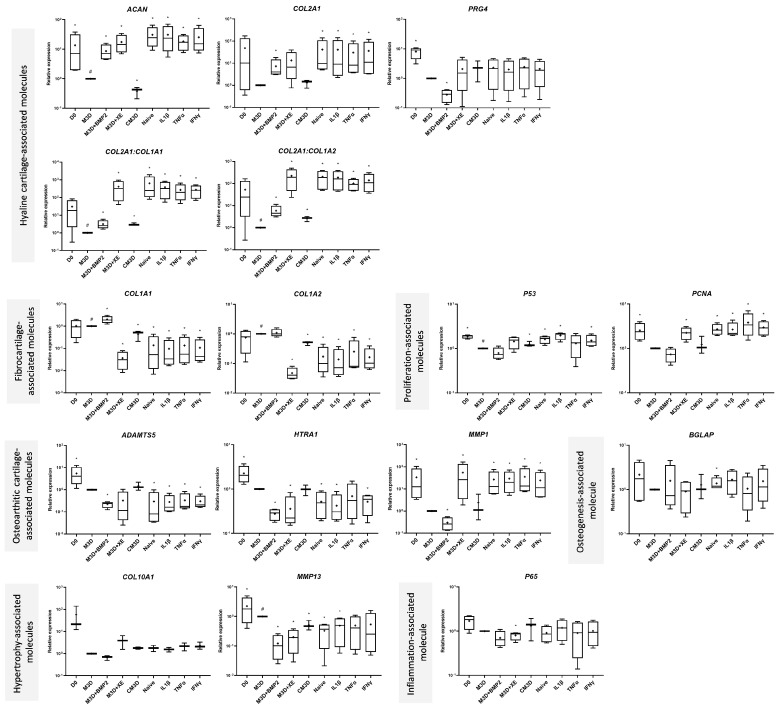
ExoEasy elution buffer is not a suitable exosome vector because it affects eAC gene expression of cartilage- and OA-associated markers. After 24 h priming of BM-MSCs (P3) with either IL-1β, TNF-α, or IFN-γ, unconditioned medium (M3D) was conditioned for 24 h and exosomes were isolated from CMs using the MAC method. M3D was aliquoted, supplemented with fresh exosomes and stored at −80 °C. Controls were included by supplementing M3D with BMP2 (50 ng/mL) or Qiagen elution buffer (XE) and CM3D corresponds to BM-MSC culture medium conditioned for 24 h. eACs (P2) were seeded in collagen sponges at 800,000 cells/sponge and then cultured under hypoxic atmosphere with the different media for 14 days. Then, sponges were harvested, washed twice with PBS and stored at −80 °C. Total RNA was collected from these cultures and RT-qPCRs were run to assess gene expression. The expression of target genes was normalized using the reference genes *β-ACTIN* or *PPIA*. The D0 condition corresponds to eACs cultured in monolayer until P2. Experiments were repeated with different strains of eACs and BM-MSCs (*n* = 4). Values are represented as box plots (median, quartiles, extreme values, “+” corresponds to the mean) and were tested using the Mann–Whitney test, * *p* < 0.05 significantly different from the M3D condition, # *p* < 0.05 significantly different from the CM3D condition. BM-MSCs, bone marrow-mesenchymal stromal cells; BMP2, bone morphogenetic protein 2; CM, conditioned medium; eACs, equine articular chondrocytes; M3D, control medium with 2% FBS; P2, passage 2; P3, passage 3; RT-qPCR, reverse transcription-quantitative polymerase chain reaction; XE, Qiagen elution buffer.

First, NTA analysis revealed that pro-inflammatory priming strongly reduced the heterogeneity in particle diameter (Figure 7). Indeed, naive-exos exhibited coefficients of variation (CVs) of 42.72%, 6.79% for IL1β-exos, 1.91% for TNFα-exos, and 16.01% for IFNγ-exos. Moreover, BM-MSC priming tended to slightly decrease the MAC-exos concentration, although the difference was not significant (Figure 7). eACs grown in the presence of CM3D exhibited an mRNA profile similar to those presented in Figure 3 and Figure 5. Briefly, major consistent results included the decrease in *ACAN*, *COL1A1*, and *COL1A2* expression with the concomitant increase in both functional ratios, *COL2A1* and *COL10A1* mRNA levels (Figure 8). In contrast to what we observed when other eAC strains were cultured with CM3D (Figure 3 and Figure 5), *P53* levels were significantly higher than in the M3D condition, but *MMP13* mRNA levels were lower, and those of *P65* remained unchanged. Surprisingly, the elution buffer exhibited effects that were not observed in our previous experiments (Figure 5 and Figure 6). In comparison with the M3D control, the XE buffer triggered an increase in *ACAN*, *COL2A1* expression, and both functional ratios, but decreased the expression of *COL1A1* and *COL1A2*. Moreover, we did not notice any modulation of *PRG4* expression. The XE buffer tended to increase *P53* mRNA levels and significantly increased *PCNA* levels (Figure 8). When eACs were cultured with XE buffer, *ADAMTS5* and *HTRA1* transcript levels did not decrease, whereas the *MMP1* levels increased, compared with the M3D condition. We did not observe any notable modulation of *BGLAP* expression in eACs cultured in the presence of the XE buffer; however, *COL10A1* mRNA levels tended to be higher than in the M3D control. Moreover, the XE buffer also lowered the expression of the *MMP13* and *P65* markers. All the four exosome conditions—i.e., naive-, IL1β-, TNFα-, and IFNγ-exos—significantly increased *ACAN*, *COL2A1* expression, and both *COL2A1:COL1A1* and *COL2A1:COL1A2* ratios, compared with the M3D condition. Although *PRG4* expression remained unchanged, *COL1A1* and *COL1A2* mRNA were greatly diminished in eACs cultured in the presence of each type of MAC-exos. All these variations were more pronounced than those observed in the CM3D condition (Figure 8). Moreover, proliferation-associated *P53* and *PCNA* expressions were also significantly higher in these conditions, except in the case of TNFα-exos for which no statistical difference was detected. eACs cultured with MAC-exos, naive or primed, exhibited a decrease in the expression of *ADAMTS5* and *HTRA1* and an increase in *MMP1* levels, compared with the M3D condition. Interestingly, *BGLAP* expression was significantly increased only when eACs were cultured with naive- and IL1β-primed exosomes when compared with the M3D control. Similar to the eACs cultured with M3D + XE, all exosome conditions tended to raise *COL10A1* levels, while decreasing the *MMP13* expression, compared with the M3D condition. However, for this latter marker, the difference was significant only for naive- and IL1β-exos. Finally, *P65* levels remained unchanged (Figure 8).

Overall, naive or primed MAC-exos had similar effects on the eAC mRNA profile. However, we observed a strong effect of MAC elution buffer on the eAC gene expression profile that was not observed in previous experiments and concealed the effects of MAC-exos on the expression of healthy and OA cartilage genes in eACs.

### 2.7. The Presence of the Elution Buffer Also Conceals the Impact of Primed MSC-Exos on the Synthesis of Healthy and OA Cartilage-Related Markers in eACs

**Figure 9:**
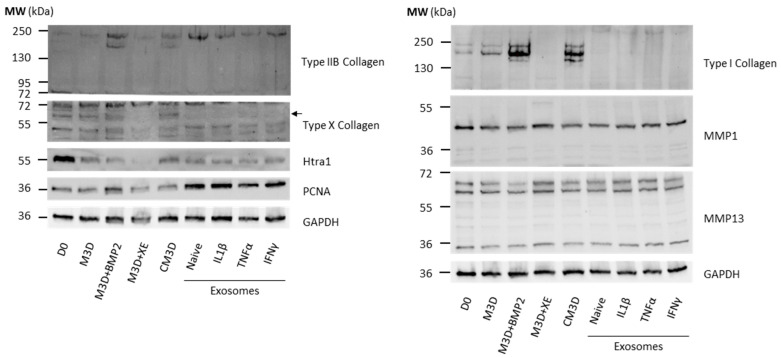
Impact of exosomes from BM-MSCs primed with pro-inflammatory cytokines on eAC expression of cartilage- and OA-associated proteins is hidden by the ExoEasy elution buffer effect. CMs from naive or primed (IL-1β, TNF-α or IFN-γ for 24 h) BM-MSCs (P3) were harvested, and exosomes were isolated using the MAC method. Unconditioned medium (M3D) was aliquoted, supplemented with fresh exosomes, and stored at −80 °C. Controls were included by supplementing M3D with BMP2 (50 ng/mL) or Qiagen elution buffer (XE) and CM3D corresponds to BM-MSC culture medium conditioned for 24 h. eACs (P2) were seeded in collagen sponges at 800,000 cells/sponge and then cultured under hypoxic atmosphere with the different media for 14 days. Then, sponges were harvested, washed twice with PBS, and stored at −80 °C. The total proteins were extracted using a dedicated buffer supplemented with protease inhibitors and Western blots were carried out to evaluate protein levels, as shown in the representative pictures. The D0 condition corresponds to eACs cultured in monolayer until P3. The arrow indicates type X collagen bands. Experiments were repeated with different strains of eACs and BM-MSCs (*n* = 4). BM-MSCs, bone marrow-mesenchymal stromal cells; CM, conditioned medium; eACs, equine articular chondrocytes; M3D, control medium with 2% FBS; P2, passage 2; P3, passage 3.

Regarding the protein amount, CM3D induced the overexpression of type I and IIB collagens compared with the M3D control (Figure 9). Moreover, no variation in type X collagen, Htra1, PCNA, MMP1, or MMP13 protein accumulation was detected. As observed at the mRNA level (Figure 8), the XE buffer displayed stronger effects on the eAC phenotype than observed previously. The XE buffer seemed to prevent type I and X collagen and Htra1 accumulation. However, in comparison with the M3D control, XE buffer tended to lead to slightly increased MMP13 protein amounts. Consistent with what we observed at the mRNA level (Figure 8), eACs cultured with naive or primed MAC-exos did not exhibit any changes in type I and X collagen protein amounts, similar to that observed when eACs were grown in the presence of the XE buffer. On the contrary, type IIB collagen and PCNA protein amounts were increased in all the MAC-exos conditions, compared with the M3D and M3D + XE conditions. Furthermore, Htra1 protein amounts were higher in eACs cultured with MAC-exos, reaching levels comparable to those obtained in the M3D condition. eACs cultured with exosomes had similar MMP1 and MMP13 protein amounts compared with the M3D + XE condition.

Altogether, these results demonstrated that the MAC elution buffer exerts a substantial impact on healthy and OA cartilage-associated gene expression of eACs but also, to a lesser extent, on their protein expression profile (Figure 8 and Figure 9). The fact that this effect was only observed in the latter experiments lies in the MAC isolation methodology. Due to lower exosome concentration in MAC isolates in the assays presented in Figure 8 and Figure 9, we had to add a higher volume of exosome-containing XE buffer to reach 6 µg of total proteins per collagen sponge (Table S1). Therefore, eACs were cultured in higher XE buffer concentrations than in previous experiments (Figure 3, Figure 4, Figure 5 and Figure 6), leading to a more pronounced effect of this reagent.

### 2.8. Additional UF Step after MAC Purification Reveals the Effect of Naive and Primed Exosomes on the eAC Phenotype

**Figure 10:**
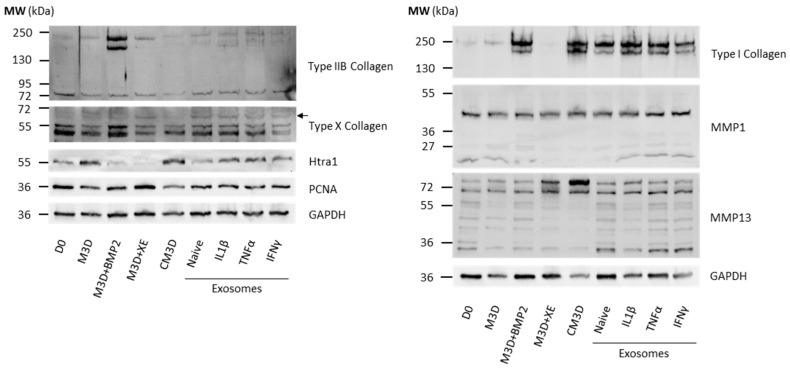
Purification method changes the effect of exosomes from BM-MSCs on eAC expression of cartilage- and OA-associated proteins. CMs from naive or primed (IL-1β, TNF-α or IFN-γ for 24h) BM-MSCs (P3) were harvested, and exosomes were isolated using the MAC + UF method. Unconditioned medium (M3D) was aliquoted, supplemented with fresh exosomes and stored at −80 °C. Controls were included by supplementing M3D with BMP2 (50 ng/mL) or Qiagen elution buffer (XE) and CM3D corresponds to BM-MSC culture medium conditioned for 24 h. eACs (P2) were seeded in collagen sponges at 800,000 cells/sponge and then cultured under hypoxic atmosphere with the different media for 14 days. Then, sponges were harvested, washed twice with PBS and stored at −80 °C. The total proteins were extracted using a dedicated buffer supplemented with protease inhibitors and Western blots were carried out to evaluate protein levels, as shown in the representative pictures. The D0 condition corresponds to eACs cultured in monolayer until P3. The arrow indicates type X collagen bands. Experiments were repeated with different strains of eACs and BM-MSCs (*n* = 4). BM-MSCs, bone marrow-mesenchymal stromal cells; CM, conditioned medium; eACs, equine articular chondrocytes; M3D, control medium with 2% FBS; P2, passage 2; P3, passage 3.

To eliminate the elution buffer from the MAC-exos solution, we repeated the same experiments as those presented in Figure 8 and Figure 9, but with the addition of a UF step after MAC purification. For this last part of the study, eACs cultured in the presence of elution buffer exhibited a very similar protein expression profile as that obtained in Figure 9, supporting these previous results (Figure 10). Conversely to the results presented in Figure 9, naive and primed exosomes ceased to follow M3D + XE trends. When compared with the naive-exos, IL1β- and TNFα-exos showed increased accumulation of type I and II B collagen protein amounts. However, when eACs were cultured with IFNγ-exos, type I and IIB collagen protein levels were similar to those observed in eACs cultured with naive-exos. No modulation of type X collagen and PCNA was observed in comparison with the M3D control, but the levels of these two proteins were higher when eACs were cultured with exosomes than with CM3D (Figure 10). Nevertheless, in comparison to the M3D condition, MAC-exos showed decreased Htra1 expression, with a stronger effect of both naive- and IFNγ-exos. Further, eACs cultured with primed MAC-exos exhibited higher levels of Htra1 compared with naive-exos. Compared with the naive-exos condition, MMP1 levels were not modified, except after eAC treatment with TNFα- and IFNγ-exos, which slightly increased the accumulation of this protein. Finally, regardless of the priming method, MAC-exos seemed to induce a slight increase in MMP13 expression in comparison with M3D (Figure 10).

The addition of a UF step after the MAC exosome purification, employed in an attempt to eliminate the presence of the elution buffer, mitigates its influence on eAC phenotype. By implementing this modification to the isolation protocol, we were able to accurately investigate the impact of purified exosomes on the expression of healthy and OA cartilage-associated proteins by eACs. All naive and primed exosomes demonstrated an interesting modulation of the eAC protein profile. All types of exosomes increased collagen accumulation and decreased Htra1 levels, especially IL1β- and TNFα-exos. These specificities make each type of exosome attractive and promising in the context of hyaline cartilage regeneration and OA management.

## 3. Discussion

OA is a degenerative disease affecting cartilage in humans and horses. Characterized by progressive cartilage breakdown, it leads to lameness, reduced joint function, increased injury risk, and animal welfare concerns. OA affects horses of all ages and can lead to premature career termination and substantial economic consequences. Current OA treatments only focus on symptom relief and reduction in OA progression, but there is currently no therapy capable of regenerating functional hyaline cartilage. MSC-based therapies have shown promising results in OA management due to the anti-inflammatory, immunomodulatory, and regenerative properties of these cells. Nevertheless, limitations inherent to cell therapies lead to effectiveness and biosafety concerns that prevent the clinical application of these strategies. In the last few years, numerous studies have provided compelling evidence regarding the involvement of the secretome in the MSC therapeutic potential, making this biological material a promising tool for innovative OA therapies. As part of this effort, two studies led by our research team recently demonstrated that the equine BM-MSC secretome can increase eAC migration and collagen synthesis [21]. Moreover, we were able to modulate this therapeutic potential by stimulating BM-MSCs with equine pro-inflammatory cytokines (IL-1β, TNF-α, and IFN-γ) [24]. Despite these encouraging results, it is now recognized that the use of exosomes instead of the whole secretome ensures better safety, prevents some standardization issues, and offers valuable targeting capacities [25,26,27]. In this context, our study aimed to experiment, adapt, and compare several common purification methods to isolate exosomes from MSC-CM. Additionally, following the logical progression of our previous studies, our work aimed to assess whether exosomes derived from naive or primed MSCs could improve the chondrocyte phenotype and/or cartilaginous ECM synthesis as well as the whole MSC secretome does.

### 3.1. MSC-Exos Isolation and Characterization

In this study, we aimed to identify the best purification method to isolate MSC-exos with satisfactory yield and purity levels, while preserving their functional properties. MAC and SEC are both common and well-documented purification techniques that have specific advantages. On the one hand, MAC provides a large quantity of exosomes, but with moderate purity; on the other hand, SEC provides highly purified exosomes, but at a lower yield. Moreover, SEC is based on the separation of different particles only according to their diameter. Therefore, SEC isolates may also contain co-isolated particles that are not EVs (e.g., low-density lipoproteins present in FBS [35]), a limitation partially overcome in the MAC method [29]. Nevertheless, Qiagen does not provide any precise information on the underlying purification principle, only giving the following details: “The method does not distinguish EVs by size or cellular origin, and is not dependent on the presence of a particular epitope. Instead, it makes use of a generic, biochemical feature of vesicles to recover the entire spectrum of extracellular vesicles present in a sample” [36].

The first part of this study demonstrated that both MAC (ExoEasy Maxi kit; Qiagen) and SEC (qEV columns; Izon) purification strategies can effectively isolate equine MSC-exos. We combined TEM, NTA and Western blot approaches to ensure the presence of intact exosomes in isolates. TEM pictures revealed a significant presence of “contaminants” in MAC-exos isolates compared with CM3D and SEC-exos. This result is consistent with the larger mean particle diameter observed in NTA analyses, which has been previously reported in the literature on EVs extracted from human plasma [29]. The most plausible explanation is that these impurities originate from the Qiagen elution buffer, which may also have reacted with the TEM fixation reagent or aggregation during the freezing step at −80 °C. After a literature review, we observed that most studies focus only on detecting a few exosome markers, such as CD9, CD63, and CD81 tetraspanins, to confirm the presence of exosomes in samples. However, according to ISEV recommendations, it is essential to demonstrate the presence of at least one surface and one intraluminal exosomal protein, along with a “contaminant” marker to evaluate the purity of the isolated exosomes [34]. Here, we set up a large panel of exosomal markers. To the best of our knowledge, only one study performed in horses has used such a comprehensive panel of markers [37]. Prior to our study, only a few antibodies were known to detect equine exosome proteins, which made exosome characterization laborious. In this study, we identified a panel of antibodies to properly characterize a panel of exosome markers. However, we were unable to find an antibody compatible with equine CD82 and thus designed a custom antibody from peptide immunization to hybridoma selection. For most exosome markers, spot intensity was the highest for MAC, followed by PEG precipitation and SEC. This detail highlights the difference in yield between the three purification methodologies. MAC pre-eminence regarding exosome yield is also supported by the higher particle concentration in MAC samples than in SEC. Nevertheless, BSA staining was significantly lower for SEC-processed samples compared with either of the other methods, supporting the use of SEC to collect highly purified EVs. These data endorse those presented in a recent study that highlighted both that using MAC leads to a higher yield, but lower purity levels compared with SEC [29]. As a result, both MAC and SEC are valid strategies to collect purified exosomes. Our Western blot assays also revealed an interesting doublet pattern for the Alix protein. Given the observed molecular weight shift of up to 20 kDa, the most plausible hypothesis is a post-translational modification, such as glycosylation, methylation, or sumoylation [38]. Interestingly, this change in molecular weight has already been demonstrated in a study carried out on exosomes derived from murine neuroglial cells [39], suggesting that this specificity is not associated with either the equine origin of the exosomes or the cell type used in our study. Moreover, this study appears to suggest that exosome content uptake does not predominantly occur through direct fusion with the cell membrane, but rather through endocytosis. The PKH26 assays showed red spots with a diameter of several µm, mainly found in the perinuclear region. These spots, too large to be individual exosomes, probably correspond to groups of exosomes labeled with PKH26 in endosomes or endolysosomes. Accordingly, after internalization, exosomes have been shown to undergo transport to endosomes, which, during the maturation process, can fuse with lysosomes, resulting in the formation of endolysosomes [40]. This pathway leads to the degradation and recycling of exosomal proteins, nucleic acids, and lipids, facilitated by the acidic and enzymatic components present within the lysosomes. Furthermore, immunofluorescent staining assays also revealed the presence of a slight PKH26 signal in eACs treated with M3D-exos. Despite multiple ultracentrifugation steps, it is known that FBS considered as exo-depleted (or sometimes “exo-free”) still contains a noteworthy concentration of small EVs [41]. This signal with M3D-exos may also be due to residual PKH26 micelles created during the labeling process.

### 3.2. Comparison between MAC and SEC Purification Strategies

Herein, we isolated MSC-exos using both MAC and SEC approaches and compared their potential to modulate the gene and protein expression of common healthy and pathological cartilage markers by eACs cultured as organoids. These results emphasized that MAC-exos induced a more substantial enhancement of the eAC hyaline-like phenotype compared with SEC-exos, proportional to the exosome concentration. We demonstrated that MAC-exos better favored the hyaline phenotype of eACs by modulating the accumulation of healthy and OA cartilage-associated markers in comparison with SEC-exos. Nevertheless, due to the significantly lower yield obtained with the SEC isolation method, we were compelled to use different concentrations of MAC- and SEC-exos when treating eACs (i.e., 12 µg/sponge for 2X MAC-exos and 1 µg/sponge for 2X SEC-exos). It is noteworthy that yield measurements were based on the total protein concentration of the exosome isolates. Nevertheless, MAC-exos exhibited much lower purity levels than SEC-exos, as indicated by a higher proportion of BSA. Consequently, the exosome concentration in the MAC-exos preparation was overestimated in the samples. The estimation of exosome concentration using the NTA analysis also has its caveats, because the presence of protein/particle aggregates can lead to inaccurate measurements. This statement emphasizes the urgent need for a reliable method to properly and specifically quantify the exosome concentration, which will also be mandatory for their therapeutic use.

The reason that MAC-purified exosomes lead to a better improvement of eAC phenotype than SEC-purified EVs may lie in sample purity. Demonstrating higher purity levels, SEC-exos isolates contain a higher proportion of exosomes than MAC-exos samples, illustrated by a higher particle-to-protein ratio [29]. Apart from EVs, the secretome of MSCs also contains a wide variety of soluble elements that demonstrate biological activity and can bind to the surface of exosomes [42]. The formation of this structure, known as protein corona, depends on physicochemical parameters (particle diameter, curvature, charge, etc.) and the protein composition of the particle’s surface enabling specific interactions. Obviously, the purification step has a direct impact on the size, composition, and function of exosomes. For example, a recent study demonstrated that protein corona removal from human placental-expanded stromal cell-derived EVs by SEC or ultracentrifugation abrogated their angiogenesis, regenerative, and immunomodulatory potential [43]. Our study found that SEC-exos exhibited higher purity than MAC-exos. Consequently, it is plausible to hypothesize that the corona of MAC-exos contains a greater abundance of bound soluble elements, which likely contribute significantly to the functional role of MAC-exos. This effect was observed by comparing the effect of the CM3D and MAC-exos on the protein accumulation of type I collagen. Indeed, the increase in type I collagen was higher in eACs cultured with CM3D than with MAC- or SEC-exos. This is not the first demonstration of a stronger impact of the MSC secretome compared with purified exosomes [44]. Even though the type I collagen is associated with fibrocartilage, its increase with CM3D suggests the stimulation of MEC neosynthesis which, despite properties differing from hyaline cartilage, is still preferable to the absence of tissue. However, MSC-exos interestingly limited the accumulation of type I collagen in eACs while promoting a greater increase in type II collagen protein levels compared with CM3D, resulting in a more hyaline-like tissue. This increase in collagen accumulation can potentially be attributed to the decrease in Htra1 protein levels after adding MAC-exos on cartilaginous organoids.

### 3.3. ExoEasy Elution Buffer Composition

During this study, we faced a major unexpected outcome with the Qiagen elution buffer used for MAC exosome isolation. Indeed, MAC-exos induced promising effects on eAC phenotype in comparison with the M3D + XE control in the first experiments. Moreover, the addition of a UF step following exosome isolation by MAC did not initially result in any notable modification in the impact of these particles on eACs. These results logically suggested that MAC was suited for exosome purification; however, in the next set of experiments, we highlighted that the XE buffer induced a significant modulation of the eAC gene and protein expression profile. As previously mentioned, the disparity between these series of experiments can be explained by the different proportions of XE buffer in the M3D employed to culture eAC organoids, which was higher for the later assays. The noteworthy effects included a strong increase in *ACAN* and *PCNA* expression, along with the decrease in *COL1A1*, *COL1A2*, *ADAMTS5*, *HTRA1*, *MMP13,* and *P65* mRNA levels. Regarding the protein profile, the XE buffer strongly decreased the type I, type X collagens, and Htra1 accumulation. These observations raise interest regarding the potential therapeutic value of the Qiagen elution buffer in the context of cartilage regeneration. Nevertheless, the manufacturer has not disclosed any details regarding the composition of this buffer, which makes complex the identification of elements of interest [36]. Furthermore, the ExoEasy kit is widely acknowledged for its versatility in various applications such as NTA and TEM, or DNA, RNA, and protein extraction. However, although the manufacturer explicitly mentions that MAC is suitable for the physical and biological characterization of exosomes, there is no recommendation for a functional or therapeutic application of MAC-exos [36]. Several investigations have explored the impact of exosomes purified using the Qiagen ExoEasy kit on recipient cells [45,46,47]. Nevertheless, to the best of our knowledge, no previous experiments have considered the potential effect of the XE buffer by adding a XE control, as we did. The effect of the XE buffer raises substantial concerns that could call into question the relevance of the therapeutic use of exosomes purified only by MAC and, consequently, the results presented in these studies. Additional research is warranted to ascertain the effect of the Qiagen elution buffer on multiple cell types, and especially in the context of equine OA, on the phenotype of eACs.

### 3.4. The Potential of Pro-Inflammatory Priming on the Therapeutic Effect of Exosomes from Equine BM-MSCs

The therapeutic interest of exosomes derived from MSCs from different tissue sources has been demonstrated in the treatment of various pathological conditions including liver, kidney, cardiovascular, and immune-related diseases [48]. Regarding cartilage regeneration and OA management, exosomes derived from BM-MSCs can increase the expression of cartilage ECM components and reduce inflammatory-related markers [49]. Nevertheless, the therapeutic properties of the MSC secretome can be enhanced by various external stimuli, such as pro-inflammatory cytokines. As an example, umbilical cord (UC)-MSCs primed with transforming growth factor β (TGF-β)-secreted EVs can upregulate chondrogenesis markers (*COL2* and *ACAN*) and downregulate the expression of fibrocartilage-associated markers (*COL1* and *RUNX2*) in chondrocytes [50]. Herein, we showed that exosomes derived from BM-MSCs primed with equine IL-1β, TNF-α, or IFN-γ present different modulation levels of the eAC phenotype. IL1β- and TNFα-exos tended to induce a stronger increase in type I and IIB collagens accumulation than naive- and IFNγ-exos, while also increasing Htra1 levels. This observation suggests the diversity of the molecular pathways associated with the pro-inflammatory stimulation of MSCs, confirming our previous results on CMs derived from primed BM-MSCs [24]. Because a single cytokine does not recreate the whole pro-inflammatory environment that occurs in OA joints, associating several immunomodulatory markers could potentialize their priming potential and improve MSC-exos therapeutic capacities. The presence of multiple cytokines in different proportions has been shown to lead to the modulation of the MSC phenotype compared with single molecule priming, along with different MSC secretory profiles [51]. Therefore, the therapeutic capacities of MSC-exos can potentially be tuned to suit the purpose of their use.

### 3.5. Toward Mass Production

Once the therapeutic potential of exosomes has been thoroughly evaluated, the ultimate goal is to facilitate their large-scale production for extensive application. Nevertheless, our work highlights the difficulty of obtaining reproducible effects with cells originating from different individuals. Our experiments were carried out on different strains of MSCs (for exosome collection) and eACs (for sponge seeding) that have different properties and, as a consequence, variable responses according to the age and condition of the donor’s health [52,53]. These consequences are illustrated through the slight modulation of eAC gene and protein expression profiles obtained for CM3D and MAC-exos conditions in MAC vs. SEC experiments and MAC vs. MAC + UF experiments. Ensuring reproduction of the exosome effects is fundamental to guaranteeing the biosafety of their use and a stable therapeutic outcome. A solution to this worrisome issue may lie in pro-inflammatory priming. In particular, previous studies have demonstrated that the functional variations observed among clones originating from murine MSC subpopulations can be mitigated through priming with IFN-γ and TNF-α [54]. Consistent with these findings, our results revealed higher uniformity in particle diameter following stimulation of BM-MSCs with IL-1β, TNF-α, or IFN-γ. In the context of large-scale production, it may also be worthwhile to identify a specific MSC strain to produce exosomes, and then to characterize and use them as therapeutics. However, this option excludes the possibility of personalized therapy. Turning to immortalized MSCs may also be an appealing alternative to obtain a reproducible therapeutic effect, while also granting the ability to expand these cells indefinitely across numerous passages [55,56]. Immortalized MSCs also raise several concerns regarding potential tumorigenicity risks; however, the collection and therapeutic use of their purified exosomes may possibly overcome this limitation.

Moreover, the prospective implementation of the production and purification methods used in this study for clinical applications on a larger scale is likely to encounter an additional limitation. Indeed, the purification method used in these assays included a UF step, which can result in a significant loss of total exosomes through adhesion to the filter. To account for this loss, we had to condition three times the amount of medium compared to the MAC methodology and, thus, use three times the number of consumables and reagents. Further, although MAC is less time-consuming than SEC or ultracentrifugation protocols for exosome isolation, it still requires a significant amount of time, which raises concerns about its suitability for large-scale applications. To address both the cost and time issues associated with the therapeutic use of exosomes, innovative mass production methods coupled with purification systems are currently in development. For example, bioreactors offer an interesting opportunity to culture a large amount of cells that can secrete a significant volume of exosomes [57]. Additionally, using these dynamic 3D culture approaches may faithfully mimic in vivo conditions, surpassing the limitations of conventional 2D expansion systems. Among others, this advancement has the potential to facilitate the clinical application of exosome-based strategies, and also enhance the therapeutic efficacy of exosomes derived from equine BM-MSCs.

## 4. Materials and Methods

### 4.1. Cell Isolation and Culture

BM-MSCs were isolated from horses aged between 2 and 13 years old, as detailed in our previous publications [58,59,60,61]. Above this age, there is a risk of collecting cells altered by aging and, as a consequence, providing reduced therapeutic capacities. Bone marrow was harvested by sternal puncture and underwent gradient centrifugation with Ficoll-Paque PREMIUM (GE Healthcare Bio-Sciences; Chicago, IL, USA). The interphase was seeded in an isolation medium (Low Glucose-Dulbecco’s Modified Eagle’s Medium (LG-DMEM); Invitrogen Life Technologies; Carlsbad, CA, USA) containing 30% FBS (Invitrogen Life Technologies), 10^-7^ M dexamethasone (Sigma-Aldrich) at 37 °C under a 5% CO_2_ and 95% humidity atmosphere. Amplification was carried out for several days until the formation of MSC colonies. After trypsinization (Invitrogen), MSCs were reseeded at 5 × 10^3^ cells/cm^2^ (passage P1) and amplified in an MSC amplification medium corresponding to LG-DMEM containing 20% FBS and supplemented with antimicrobial agents (100 IU/mL penicillin, 100 µg/mL streptomycin, and 0.25 µg/mL amphotericin B (PSA); Eurobio Scientific; Les Ulis, France). Then, isolated MSCs were characterized through an assessment of their proliferative capacities, multipotency, the presence of surface markers (CD29, CD44, CD73, CD90, CD105), and the absence of CD45 and type II major histocompatibility complex (MHC).

eAC isolation was performed on cartilage pieces collected from carpal and femoral condyles of 3- to 10-year-old horses (various breeds and types). Samples were sliced into fragments a few millimeters thick and incubated at 37 °C for 45 min with 2 mg/mL Streptomyces griseus protease (Sigma-Aldrich), then rinsed with PBS and re-incubated for 18 h with 2 mg/mL Clostridium histolyticum type I collagenase (Invitrogen Life Technologies). Both of these enzymes were diluted in High Glucose-Dulbecco’s Modified Eagle’s Medium (HG-DMEM; Invitrogen) supplemented with 1% PSA. After digestion steps, cell suspensions were filtered using a 70 μm nylon strainer, cells were counted, aliquoted, and stored in liquid nitrogen or directly reseeded (P0) at 2 × 10^4^ cells/cm^2^ in HG-DMEM containing 10% FBS and 1% PSA.

All methods and procedures described in the present study were approved by the ethics committee “Comité d’éthique/Agence nationale de sécurité sanitaire/École nationale vétérinaire d’Alfort/Université Paris-est Créteil” (ComEth Anses/ENVA/UPEC) (Date of approval: 10 March 2015, permit number: 10/03/15-12).

### 4.2. Priming and Medium Conditioning

BM-MSCs were cultured in MSC amplification medium until they reached 60% confluency. After two PBS washes, cells were incubated with preconditioning medium (LG-DMEM supplemented with PSA and 20% exosome-depleted FBS (exo-free FBS; Gibco, Grand Island, NY, USA)) for 24 h at 37 °C in a 5% CO_2_ atmosphere. When experiments required cytokine priming, IL-1β (20 ng/mL), TNF-α (10 ng/mL), or IFN-γ (100 ng/mL) was added to the preconditioning medium. The optimum cytokine concentrations were defined in our previous work [24]. Then, BM-MSCs were rinsed twice with PBS and HG-DMEM supplemented with PSA and 2% exo-free FBS (M3D or conditioning medium or unconditioned medium) was added to the cell cultures. After 24 h of conditioning, CM was collected and centrifuged once at 300× *g* for 10 min at room temperature (RT). The supernatant was then centrifuged at 3000× *g* for 20 min at RT and filtered on 0.22 µm (CM3D). Part of the CM3D was aliquoted and stored at −80 °C and exosomes were isolated from the remaining CM following the different methods detailed below.

### 4.3. Exosome Isolation with Polyethylene Glycol

To isolate exosomes from CM3D, one volume of a PBS solution containing 2% protamine sulfate (PS; Thermo Fisher Scientific; Waltham, MA, USA) and 50% polyethylene glycol (PEG) 35,000 (Sigma Aldrich) were added to four volumes of CM3D. After 10 min of gentle agitation and 1 h of incubation at 4 °C, the mixture was centrifuged at 12,000× *g* for 10 min at 4 °C, and the supernatant was discarded. The pellet was re-suspended in PBS for further experiments.

### 4.4. Exosome Isolation by Membrane Affinity

Exosomes were isolated from CM3D using the ExoEasy Maki kit (Qiagen, Hilden, Germany) according to the manufacturer’s guidelines. Briefly, XBP buffer was added to CM3D and the CM3D/XBP mix was entirely filtered on supplied columns through 1 min-long 500× *g* centrifugations at RT. Membranes were washed with XWP buffer and membrane-bound particles were eluted with XE buffer. Exosomes solubilized in elution buffer were used to supplement the eAC culture medium (“ExoEasy” conditions). For some experiments, membrane affinity exosome isolation was followed by a UF of the exosome/XE mix—PBS added for a final volume of 4 mL—using the Amicon Ultra-4 Centrifugal Filter 100 kDa MWCO (Millipore Sigma, Burlington, MA, USA) at 4000× *g* until 500 µL of liquid was left in the upper compartment. This step was repeated three times. The remaining solution was collected, and the filter was rinsed twice with 150 µL of PBS to remove residual particles from the Amicon membrane and the exosome solution added for a final volume of 800 µL. Exosomes solubilized in PBS were used to supplement the eAC culture medium (MAC + UF-exos).

### 4.5. Exosome Isolation by Size-Exclusion Chromatography

Before isolation, CM3D was concentrated by UF on the Amicon Ultra-15 Centrifugal Filter 100 kDa MWCO (Millipore Sigma) at 4000× *g* until 2–3 mL was left in the upper compartment. The remaining liquid was collected, the filter was rinsed twice with 1 mL of PBS, and PBS was added for a final volume of 10 mL. Exosomes were isolated from CM3D using the Automatic Fraction Collector (AFC; Izon Science) according to the manufacturer’s recommendations. Briefly, the enriched exosome solution was fractionated using a second generation qEV10/35 nm column (Izon Science). The 15 mL fraction range previously identified as containing exosomes was collected and concentrated at 1000× g using the Amicon Ultra-4 Centrifugal Filter 100 kDa MWCO (Millipore Sigma). The remaining solution was collected and the filter was rinsed twice with 150 µL of PBS to remove residual particles from the Amicon membrane and then to obtain a final volume of 800 µL of exosome solution. These exosomes solubilized in PBS were used to supplement the eAC culture medium (“qEV” conditions).

### 4.6. Exosome Protein Extraction and Assay

Unlike SEC exosome solutions (Izon), samples processed through precipitation and membrane affinity isolation (Qiagen) were previously diluted to 1:5 in PBS. Then, 50 µL of each raw or diluted sample was mixed with 50 µL of lysis buffer (radioimmunoprecipitation (RIPA)-lysis buffer (50 nM Tris-HCl, 150 mM NaCl, 1 mM NaF, 1 mM egtazic acid (EGTA), 0.25% Na-deoxycholate, 1% NP-40). After 10 min incubation at 4 °C, the protein concentration of samples collected from each of three exosome isolation protocols was measured using the Micro BCA™ Protein Assay Kit (Thermo Fisher Scientific, Waltham, MA, USA). Diluted bovine serum albumin (BSA) standards and working reagent (WR) were prepared using the manufacturer’s instructions. Standards and samples were tested in duplicate in a 96-well plate, 150 µL of WR was added to each well, and the plate was incubated at 37 °C for 1 h. The absorbance at 562 nm was then analyzed using a microplate reader (Spark 10M; TECAN^®^, Lyon, France) and the protein concentration of exosome samples was deduced from the linear regression of the standard.

### 4.7. Exosome Characterization

To characterize exosome makers, and for each isolation method, protein extracts from M3D and CM3D—which represents a 75-fold concentration—were mixed with a loading buffer (4× Laemmli Sample Buffer (Bio-Rad, Hercules, CA, USA) supplemented with 10% of β-mercaptoethanol), denatured at 95 °C for 5 min, and analyzed by Western blot.

### 4.8. Exosome Medium Supplementation

After isolation, exosomes recovered using the MAC (ExoEasy) or SEC (qEV) methods were added into M3D filtered at 0.22 µm and stored at −80 °C until use with eAC 3D culture. To correspond to the exosome quantity present in the initial CM3D, the exosome protein quantity added to the M3D was determined for each isolation method. Thereby, 6 µg and 0.5 µg of protein extract, isolated by the ExoEasy and qEV method, were added to each mL of M3D.

### 4.9. eAC Three-Dimensional Culture

After eACs (P1) reached confluency, cells were rinsed twice with PBS, trypsinized and counted. eACs were then seeded in collagen sponges manufactured by Symatèse Biomatériaux as previously described [62]. Briefly, each sponge was seeded with 8 × 10^5^ cells, incubated in normoxia at 37 °C. The next day, sponges were transferred into 24-well culture plates with M3D or corresponding media supplemented with EVs (1 mL/sponge). eACs were cultured at 37 °C under a hypoxic atmosphere (3% O_2_) and media were changed twice a week. After 14 days, sponges were collected, rinsed twice in PBS, and stored at −80 °C until further experiments.

### 4.10. eAC RNA Extraction and RT-qPCR

Total RNA was extracted from cartilaginous organoids with the RNA-Solv^®^ reagent (Ozyme, Saint-Cyr-l’École, France) according to the manufacturer’s guidelines. After total RNA quantification and DNase treatment (DNase I; Thermo Fisher Scientific, Waltham, MA, USA), total RNA (1 µg) was retrotranscribed into cDNA using the iScript™ Reverse Transcription Supermix (Bio-Rad) following the supplier’s instructions. cDNA was diluted to 1:20 in nuclease-free water and samples were stored at −20 °C until use. Real-time PCR was performed using the Go Taq^®^ Probe qPCR Master Mix (Promega, Charbonnières, France) on a CFX96 PCR detection system (Bio-Rad). Target genes and primer sequences are listed in Table 1. The 2^−∆∆CT^ method, available in the Bio-Rad CFX Manager software (3.1 version), was used to normalize results. Each gene expression was normalized using *β-ACTIN* and *PPIA* housekeeping genes.

### 4.11. eAC Protein Extraction

Cartilaginous collagen sponges were crushed at 4 °C in RIPA–lysis buffer supplemented with protease and phosphatase inhibitors (phenyl methyl sulfonyl fluoride (1 M), pepstatin A (1 µg/mL), aprotinin (1 µg/mL), leupeptin (1 µg/mL), and NA_3_VO_4_ (1 mM)) and vortexed every 5 min during 45 min. After 30 min of centrifugation at 13,000× *g* and 4 °C, supernatants were collected, total protein concentration was quantified using a Bradford assay (Bio-Rad, Hercules, CA, USA) and samples were stored at −20 °C until Western blot analysis.

### 4.12. Western Blot

Protein extracts (from eACs and exosomes) were mixed with loading buffer, denatured at 95 °C for 5 min and separated via electrophoresis on 7.5% (eAC protein extracts) and 12% (EV characterization) polyacrylamide gels (TGX Stain-Free Fast Cast Acrylamide Kit 7.5% and 12%; Bio-Rad). Proteins were transferred onto a PVDF membrane (Trans-Blot Turbo RTA Midi PVDF Transfer Kit; Bio-Rad) using a transfer system (Trans-blot^®^ Turbo™; Bio-Rad). Unspecific site blockage of PVDF membranes was carried out at RT with 10% non-fat milk diluted in TBST and membranes were incubated overnight at 4 °C with primary antibodies. After three washes of 5 min in TBST, membranes were incubated for 1 h at RT with secondary antibodies and then rinsed again with TBST. All antibodies and corresponding dilutions are listed in Table 2 for EV characterization, and in Table 3 for eAC phenotype analysis. The chemiluminescence signal (Clarity Western ECL Substrate; Bio-Rad) was detected with the ChemiDoc MP Imaging System (Bio-Rad).

### 4.13. PKH26 Labeling of Exosomes

CM3D was collected and prepared as previously detailed. After concentration using the Amicon Ultra-15 Centrifugal Filter 100 kDa MWCO (Millipore Sigma), exosome isolation was performed on the second generation of qEV original/35 nm columns using the AFC (Izon Science). PKH26 labeling was performed with the PKH26 Red Fluorescent Cell Linker Kit (Sigma Aldrich) according to the manufacturer’s instructions. Briefly, exosomes were concentrated at 4000× g using the Amicon Ultra-4 Centrifugal Filter 100 kDa MWCO (Millipore Sigma) and washed twice with diluent C. After PKH26 labeling, exosomes were concentrated in the Amicon Ultra-4 Centrifugal Filter 100 kDa MWCO, fractionated with AFC to eliminate PKH26 micelles and concentrated again. After micro-BCA quantification to determine total protein concentration, exosomes labeled with PKH26 (PKH26-exos) were stored at −80 °C until use.

### 4.14. Exosome Internalization by eACs

After eACs (P1) reached confluency, cells were rinsed twice with PBS, trypsinized, and counted. eACs were then seeded in 2D (10^4^ cells per well in 96-well plate), or 3D cultures (8 × 10^5^ cells per sponge), and incubated with M3D for 7 days at 37 °C in normoxic (2D) or hypoxic (3D) conditions. After two washes with PBS, 2D cell cultures and cartilaginous organoids were cultured in their respective incubation conditions for 24 h or 48 h with 6 µg/mL of PKH26-exos in M3D.

### 4.15. F-Actin Staining and Confocal Imaging

After treatment, 2D and 3D cultures of eACs were washed twice with PBS and fixed with 10% formalin for 30 min at 4 °C. F-actin staining was performed using XFD488 Phalloidin (AAT Bioquest, Pleasanton, CA, USA) according to the manufacturer’s instructions. Briefly, 100 μL of XFD488 Phalloidin Conjugate solution, prepared in PBS with 1% BSA, was added to sponges or wells for 90 min at RT. After three washes with PBS, samples were incubated 10 min at RT with DAPI (1 µg/mL in PBS) and washed three times again with PBS. Samples were stored at 4 °C until confocal imaging using an Olympus FV1000 confocal laser scanning (Olympus, Hamburg, Germany)

### 4.16. Nanoparticle Tracking Analysis

Nanoparticles isolated from CM were characterized using the NanoSight LM10 system (Malvern Instruments Ltd., Malvern, UK). This technique is based on the detection of nanoparticles in Brownian motion, which allows the determination of particle size and concentration. Briefly, samples were diluted with milli-Q water and passed through a 405 nm monochromatic laser beam. Then, a 60 s video recording of particles moving under Brownian motion was analyzed using the Nanosight version 3.4 software. Average nanoparticle size distribution and concentration were determined based on these videos. All measurements were carried out in triplicate.

### 4.17. Transmission Electron Microscopy

MSC-derived nanoparticle morphology and integrity were checked using negative-staining TEM. All samples were placed on a Formvar-carbon-coated copper grid (pretreated by glow discharge ELMO CORDOUAN) for 1 min at RT and negatively stained three times with 1.5%. uranyl acetate. Samples were examined using a 1011 JEOL transmission electron microscope (TEM) (JEOL USA Inc., Peabody, MA, USA) operating at 100 kV and micrographs were recorded with an Orius 200 GATAN camera.

### 4.18. Statistical Analysis

All the experiments were repeated at least three times with cells derived from different equine BM-MSC and eAC strains. The number of repetitions is indicated in the description of each experiment. Mann–Whitney U-tests were performed using the GraphPad Prism 8 software (GraphPad Software Inc., San Diego, CA, USA). A *p*-value equal or less than 0.05 was considered significant. The condition used as reference for statistical comparisons is indicated in the figure legends.

## 5. Conclusions

Of significant concern in the equine industry, OA has garnered considerable attention from research groups investigating effective methods for regenerating hyaline cartilage. In this context, there is a growing interest in MSC-derived exosomes and the development of innovative methods to exploit and improve their therapeutic use. Our study demonstrated the successful purification and characterization of MSC-exos by adapting available protocols to biological material from equine origin. Despite lower purity levels, MAC enabled the purification of a higher quantity of MSC-exos exhibiting a greater potential to improve the eAC phenotype compared with SEC. The priming of BM-MSCs with either IL-1β, TNF-α, or IFN-γ resulted in the modulation of this therapeutic effect, emphasizing the variety of pathways underlying exosome-mediated cell communication. These findings highlight the importance of considering a combination of these pro-inflammatory cytokines during MSC priming to better replicate the OA environment and achieve a more comprehensive representation of its complex characteristics. However, this work raised questions regarding the MAC elution buffer. Meant to elute exosomes isolated with the Qiagen ExoEasy kit, this solution unexpectedly demonstrated significant effects when it constituted more than 3% of the medium used for culturing eACs. Considering the objective of using MSC-derived exosomes in vivo, this observation raises concerns regarding the compatibility of the elution buffer. Nonetheless, it was possible to overcome this issue by implementing a UF step after MAC purification, effectively eliminating the need for the elution buffer.

By avoiding the limitations inherent to cell therapy while preserving the MSC therapeutic potential, the results presented in this study open up a wide variety of promising prospects and possibilities. The use of native exosomes appears to be a promising approach, and exploring the modification of these exosomes may also offer significant advantages in the context of cartilage regeneration. Emerging studies have revealed the potential for incorporating therapeutic molecules into the cargo of exosomes [63] or enhancing their targeted delivery by functionalizing their surface [64]. These advancements hold great promise not only for equine health, but also for human medicine, because both species share comparable joint physiology and mechanisms of OA development. Consequently, progress in managing OA in horses can facilitate the transfer of treatments from preclinical research to clinical medicine, aligning with the principles of the “One Health” approach.

## Figures and Tables

**Table 1:** Primer sequences for the eAC gene expression study. The shades of gray are intended to improve the reading.

Target Gene	Forward Sequence	Reverse Sequence
*ACAN*	TGT CAA CAA CAA TGC CCA AGA C	CTT CTT CCG CCC AAA GGT CC
*ADAMTS5*	AAG GGA CAC CAT GTG GCAA A	CCC ACA TGA GCG AGA ACA CT
*β-ACTIN*	GAT GAT GAT ATC GCC GCG CTC	TGC CCC ACG TAT GAG TCC TT
*COL10A1*	GCA CCC CAG TAA TGT ACA CCT ATG	GAG CCA CAC CTG GTC ATT TTC
*COL1A1*	TGC CGT GAC CTC AAG ATG TG	CGT CTC CAT GTT GCA GAA GA
*COL1A2*	CCA GAG TGG AGC AGC GGT TA	GGG ATG TTT TCA GGT TGA GCC
*COL2A1*	GGC AAT AGC AGG TTC ACG TAC A	CGA TAA CAG TCT TGC CCC ACT T
*HTRA1*	GGA CTT CAT GTT TCC CTC AA	GTT CTG CTG AAC AAG CAA CA
*MMP1*	CGA AGG GAA CCC TCG GTG GGA	TGG CCT GGT CCA CAT CTG CTC
*MMP13*	TGA AGA CCC GAA CCC TAA ACA T	GAA GAC TGG TGA TGG CAT CAA G
*P53*	CAC CTG AGG TTG GCT CTG AC	GCA CAA ACA CGC ACC TCA AA
*P65*	CAC GGA TAC CAC CAA GAC CC	GTC TGG ATG CGC TGA CTG AT
*PPIA*	CCC TAC CGT GTT CTT CGA CA	GTG AAG TCA CCA CCC TGA CA
*PRG4*	CTA CCA CCC AAC GCA ACA AA	ACT GTT GTC TCC TTA TTG GGT

**Table 2:** Antibodies used for exosome characterization by Western blot.

Antibody	Dilution	Supplier
Mouse anti-bovine serum albumin (BSA)	0.1 µg/mL	Santa Cruz Biotechnology, Dallas, TX, USA
Mouse anti-human CD9	2 µg/mL	
Mouse anti-human CD63	1 µg/mL	
Mouse anti-human CD81	0.1 µg/mL	
Mouse anti-human Alix	1 µg/mL	Covalab, Villeurbanne, France
Mouse anti-human Tsg101	1 µg/mL	
Mouse anti-equine CD82	10 µg/mL	
HRP-conjugated goat anti-mouse IgG antibody	16 ng/mL	Jackson Immunoresearch, West Grove, PA, USA

**Table 3:** Antibodies used for eAC phenotype characterization by Western blot.

Antibody	Dilution	Supplier
Rabbit anti-bovine type I collagen	1:3000	Novotec, Bron, France
Rabbit anti-human type II collagen	1:750	
Mouse anti-human type X collagen	1:1000	Sigma-Aldrich, Saint Louis, MO, USA
Rabbit anti-human GAPDH	1:3000	Santa Cruz Biotechnology, Dallas, TX, USA
Mouse anti-human PCNA	1:1000	
Rabbit anti-human Htra1	1:3000	Merck Millipore, Billerica, MA, USA
Rabbit anti-human type IIB collagen	1:750	Covalab, Villeurbanne, France
Rabbit anti-human MMP1	1:1000	Affinity Biosciences, Melbourne, VIC, Australia
HRP-conjugated goat anti-rabbit antibody	1:5000	Jackson Immunoresearch, West Grove, PA, USA
HRP-conjugated goat anti-mouse antibody	1:5000	

## Data Availability

The data are presented in this study.

## Supplementary Materials

The supporting information can be downloaded at: https://www.mdpi.com/article/10.3390/ijms241814169/s1.
