# Supplementary material for: Pro-Inflammatory Cytokine Priming and Purification Method Modulate the Impact of Exosomes Derived from Equine Bone Marrow Mesenchymal Stromal Cells on Equine Articular Chondrocytes
Source: Int J Mol Sci. 2023 Sep 16;24(18):14169. doi: 10.3390/ijms241814169 (PMC10531906; doi:10.3390/ijms241814169)
Supplement: Supplementary file 1 [file ijms-24-14169-s001.zip › ijms-2596183-supplementary.pdf]

International Journal of
Molecular Sciences

MDPI

Pro-inflammatory cytokine priming and purification method modulate the impact of exosomes derived from equine bone marrow mesenchymal stromal cells on equine articular chondrocytes

Manon Jammes[1], Frédéric Cassé[1], Emilie Velot[2], Arnaud Bianchi[2], Fabrice Audigié[3], Romain Contentin[1,†], and Philippe Galéra[1,†,*]

[1] BIOTARGEN, UNICAEN, Normandie University, 14000 Caen, France
[2] Molecular Engineering and Articular Physiopathology (IMoPA), French National Center for Scientific Research (CNRS), Université de Lorraine, F-54000 Nancy, France
[3] Center of Imaging and Research in Locomotor Affections on Equines, Veterinary School of Alfort, Goustranville, France

† These authors contributed equally to this work
* Correspondence: philippe.galera@unicaen.fr

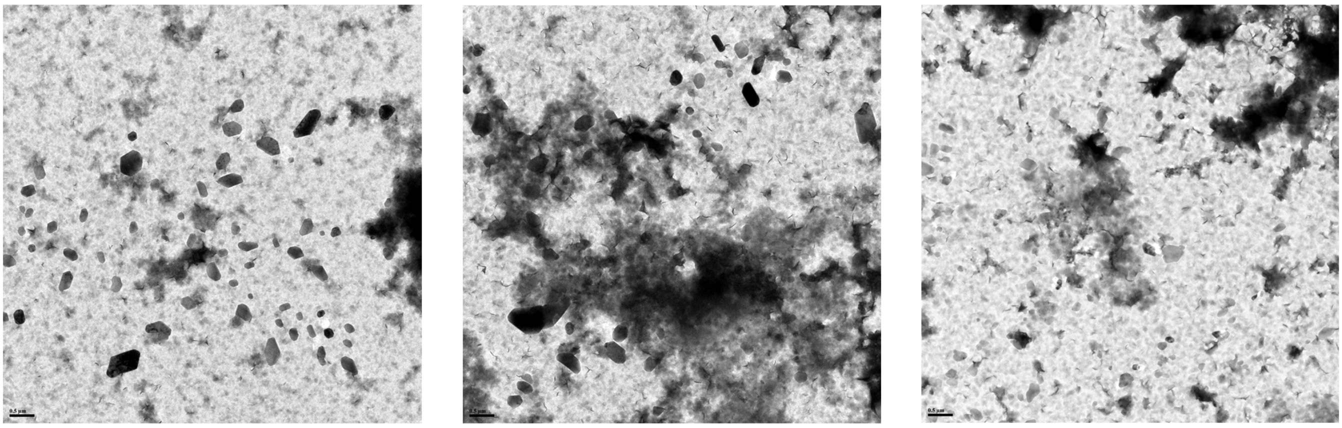

Figure S1. MAC-exos isolates contain impurities.

CM of equine BM-MSCs (P3) was collected after 24 h of culture. After centrifugation, filtration and concentration of the CMs, exosomes were purified using the MAC method (ExoEasy Maxi kit, Qiagen). Within the same day and without a freezing step, MAC-exos isolates were fixed and observed using TEM (scale bar: 0.5 µm).

BM-MSCs, bone marrow-mesenchymal stem cells; CM, conditioned media; P3, passage 3.

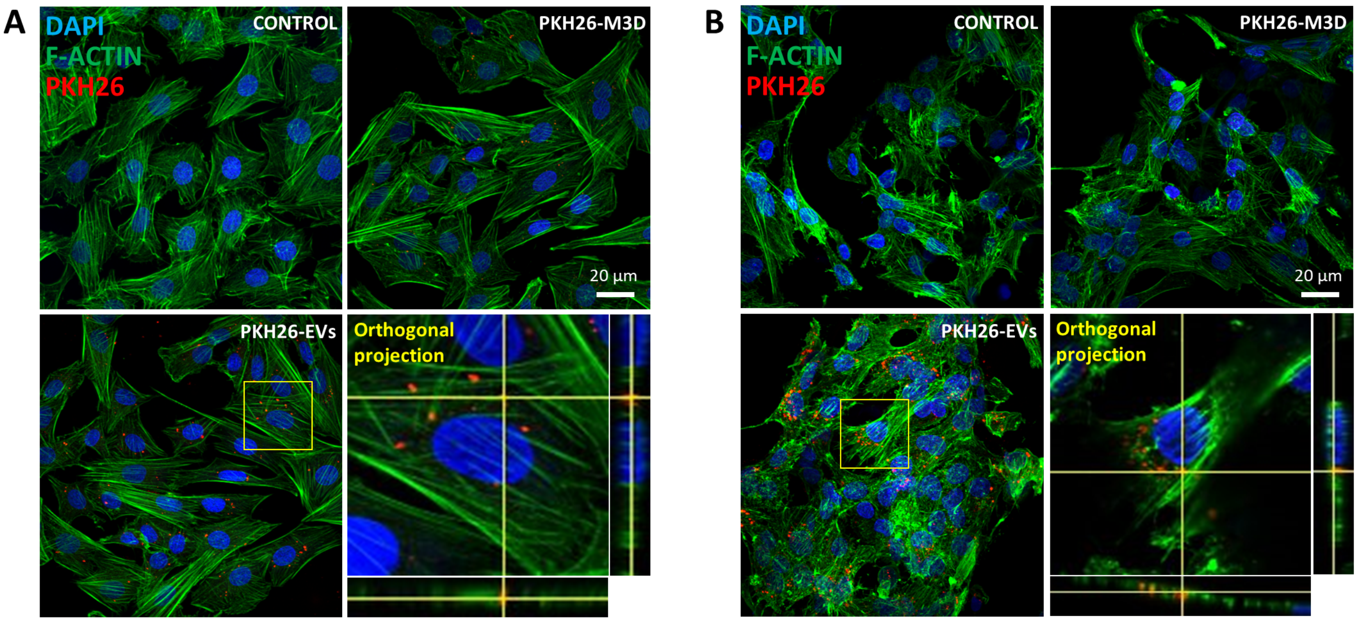

Figure S2. Exosomes derived from BM-MSCs are internalized by eACs cultured in monolayer or in collagen sponges.

CM of equine BM-MSCs (P3) was collected after 24 h of culture. After centrifugation, filtration and concentration, samples were purified on a qEV35 column using the AFC (Izon Science) and reconcentrated. Exosomes were stained with the PKH26 Red Fluorescent Cell Linker Kit (Sigma-Aldrich) and stored in PBS at -80°C until use. eAC (P2) were seeded in monolayer (**A**) at $2x10^4$ cells/cm² or in collagen sponges (**B**) at 800,000 cells/sponge, grown for 7 days under hypoxic atmosphere and treated with 6 µg/mL of PKH26-labelled exosomes. After 48 h of culture, cells were fixed, labeled with DAPI and F-ACTIN and imaged using a confocal microscope (orthogonal projection depth: 30 µm). Control condition refers to untreated cells and M3D corresponds to eACs treated with exosomes purified from unconditioned medium.

BM-MSCs, bone marrow-mesenchymal stem cells; CM, conditioned media; P2, passage 2; P3, passage 3.

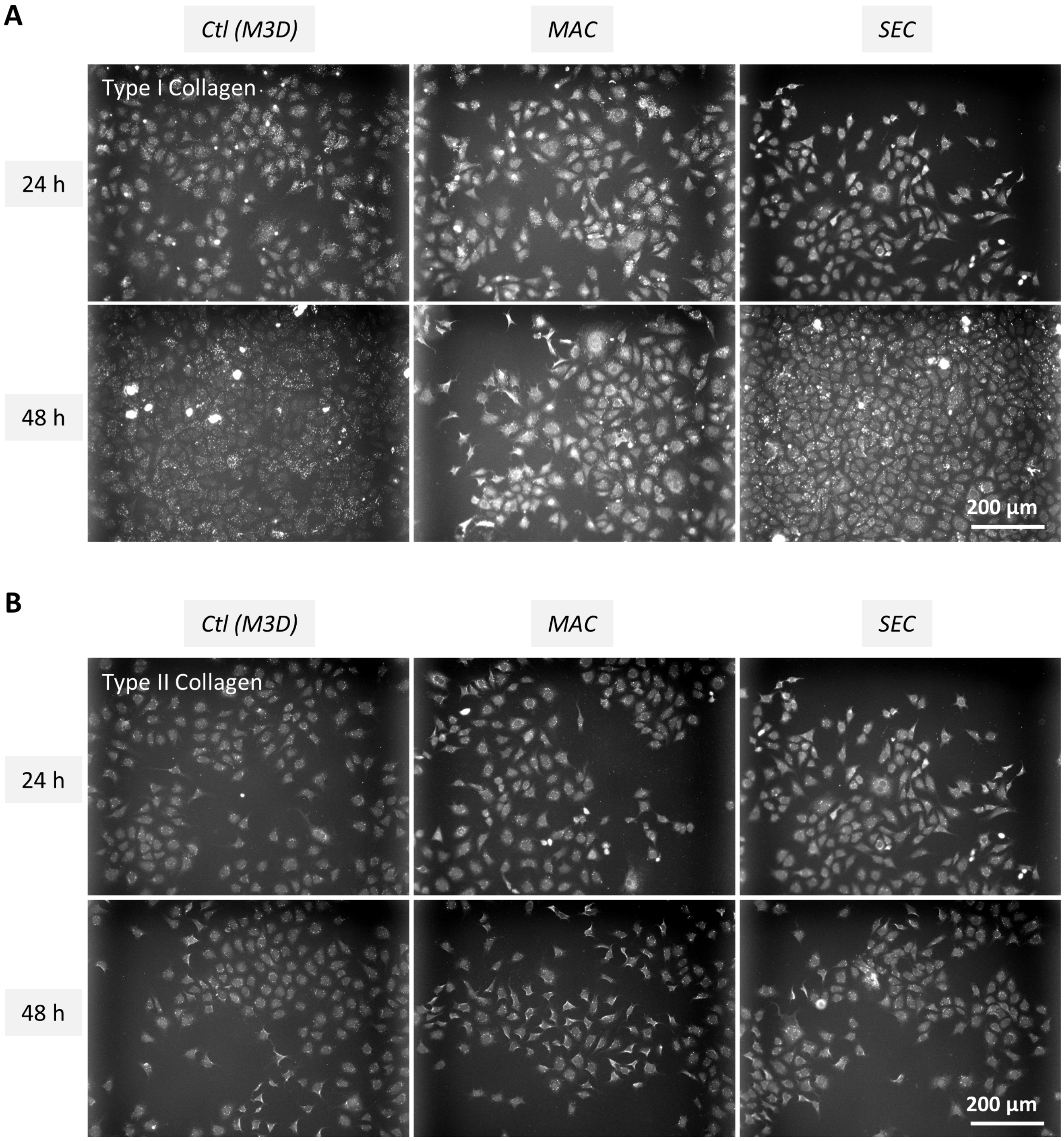

Figure S3. Immunofluorescence of the type I and II collagens in eACs exposed with MAC- and SEC-exos.

CMs from BM-MSCs (P3) were harvested and exosomes were isolated using either the MAC or the SEC method. Unconditioned medium (M3D) was aliquoted, supplemented with fresh exosomes and stored at -80°C. eACs (P2) were seeded in monolayer at $2x10^4$ cells/cm² for 24h and then cultured with the different media for 24 h or 48 h. Then, cells were washed twice with PBS and fixed. After immunofluorescence staining of type I (**A**) or II (**B**) collagen, microphotographs were taken using an IncuCyte S3 microscope. Experiments were repeated with different strains of eACs and BM-MSCs (n=4).

BM-MSCs, bone marrow-mesenchymal stem cells; CM, conditioned media; P2, passage 2; P3, passage 3.

	MAC *vs.* MAC+UF (Figures 5 and 6)	Priming MAC (Figures 8 and 9)			
		Naive-exos	IL1β-exos	TNFα-exos	IFNγ-exos
Experiment 1	0.83	8.53	8.51	5.38	6.82
Experiment 2	1.04	5.12	4.83	5.12	4.95
Experiment 3	1.45	3.87	4.19	3.38	4.38
Experiment 4	1.33	4.11	4.54	3.39	4.25
**% total volume**	**1.16**	**5.09**			

Table S1. Percentage of XE buffer used to culture eACs seeded in collagen sponges.

Regarding the total protein concentration of MAC-exos isolates, different volumes of exosomes diluted in XE buffer were added to eAC cultures. It is important to note that MAC-exos used in "MAC *vs*. MAC+UF" experiments (Figures 5 and 6) are not derived from the same BM-MSC strains as for the "Priming MAC" experiments (Figures 8 and 9).

BM-MSCs, bone marrow-mesenchymal stem cells.
